# The Relevance of Law and Economics for Fire Safety

**DOI:** 10.1007/s10694-026-01980-6

**Published:** 2026-08-28

**Authors:** Ruben Korsten, Andrea Franchini, Ruben Van Coile

**Affiliations:** Ghent University, Ghent, Belgium

**Keywords:** Fire safety, Safety regulation, Building codes, Risk, Compliance, K32, K13, L51, H41, R38

## Abstract

This article investigates how the discipline of Law and Economics (“L&E”) can guide legal frameworks for fire safety by aligning legal rules, economic incentives, and engineering practices. We first introduce core L&E concepts – externalities, information asymmetries, public-goods, and liability structures – and their relevance for fire safety. We then develop a mathematical model for the total cost of fire (for a design problem or building portfolio), and use this model to contrast prescriptive and performance-based regimes and compare three liability standards (strict liability, code-compliance negligence, and due-diligence negligence). When considering a full internalization of the cost of fire (i.e., when the private decision-maker carries all fire-related costs), our analyses show that prescriptive regimes generate over- or under-investment in preventive measures, resulting in sub-optimal societal outcomes. Performance-based regimes, on the other hand, provide private decision makers with the incentive to invest up to societally optimal safety investments. The comparison of liability regimes shows that the internalization of the cost of fire is most effectively achieved by the strict liability regime. A liability regime of negligence based on due diligence (professional standards) also pushes private decision-makers towards adopting societally optimum designs. However, when negligence is established by the (lack of) compliance with prescriptive fire safety codes, this results in societally suboptimal designs. These results imply that policymakers should favor adaptable, efficiency-oriented regulatory designs, and highlight that liability regimes should be calibrated to internalize fire safety related costs. In more general terms, the results demonstrate the power of L&E approaches for supporting discussions on fire safety legislation.

## Introduction

### Legal Frameworks in Fire Safety

Fire safety engineers play a central role in designing buildings and systems that mitigate fire risk and protect human life, property, and societal well-being. In doing so, they operate within legal frameworks and economic realities that both shape and, at times, constrain their decisions. The legal frameworks set minimum safety standards and determine liability for non-compliance, while economic realities influence the feasibility of safety measures and the incentives for stakeholders to comply with the legal framework.

The work of a fire safety engineer can be constrained by a legal framework that is overly rigid, economically inefficient, or outdated in light of modern construction techniques (e.g., use of modern construction materials, open-plan designs, and sustainable building technologies; [1, 2]). Spinardi et al. [2] and Spinardi and Law [3] note that legal frameworks often lag behind evolving building practices, creating gaps where risks accumulate unnoticed. Similarly, Bell [4] shows that rapid urbanization and high-rise growth have outpaced construction laws, leaving cities vulnerable to fire safety failures. Kodur et al. [1] add that modern construction materials and higher fuel loads amplify fire hazards in ways that building codes fail to address. Meacham [5] further emphasizes the overlooked risks in existing buildings, where poor maintenance, illegal modifications, and socioeconomic disparities heighten vulnerabilities, especially for low-income populations. These gaps highlight the need for regulatory frameworks that can allow for technological and societal changes, rather than remaining static or purely reactive and may also highlight the need for more specialized regulatory review (or systematic use of accredited third-party peer reviewers) to augment public sector expertise [6, 7]. Moreover, while most existing frameworks prioritize life safety, they often overlook broader considerations such as economic feasibility, property protection, and business continuity [8].

Historically, fire safety design has been largely prescriptive, with prescriptive rules providing exact requirements such as material choices or escape route dimensions [2]. Prescriptive rules are rarely subject to an objective evaluation of their performance, raising concerns about their real-world effectiveness. For instance, prescriptive fire safety frameworks commonly link required fire-resistance periods to building height (e.g., BS 9999 and BS 9991 in the United Kingdom, hereafter “UK”), but [9] has since challenged the validity of that proxy, highlighting that prescribed fire resistance is more appropriately linked to the likelihood and consequences of fire (lives lost and monetary losses). Furthermore, Chow [10] notes that in the Asia-Oceania regions, rapid economic development and innovative architectural designs frequently cannot comply with traditional prescriptive fire codes, indicating the need for a shift toward performance-based design (hereafter, “PBD”). The latter enables engineers to tailor safety solutions to the unique characteristics of a building while balancing regulatory compliance, design flexibility, and economic feasibility [5, 10].

Yet, the transition to PBD introduces challenges. Spinardi et al. [2] argue that PBD relies heavily on the competence of engineers and the rigor of oversight, both of which can be compromised by economic pressures. Brannigan & Smidts [11] and Brannigan [12] add that PBD often reduces complex fire scenarios to simplified "scenario fires" defined by designers, severing the link between engineering analyses and enforceable societal safety goals such as life protection. Along similar lines, Chow [10] highlights PBD’s potential misuse to cut costs, for example, through inadequate design fires that compromise safety. Spinardi [55] further notes that the expertise asymmetry between regulators and fire safety engineers limits regulators’ ability to evaluate complex PBD solutions, leading to reliance on industry-provided data and raising self-regulation risks. In environments with privatized inspections this is exacerbated according to Cook and Taylor [14], who describe a ‘climate of imperceptibility’ with PBD codes allowing developers to conceal unsafe practices. In a related field, May [15] examined the “leaky buildings” crisis in New Zealand, linking widespread weathertightness failures to the shift toward PBD combined with weak oversight and fragmented accountability. His later work argues that PBD regimes require clear performance metrics, independent verification, and enforceable sanctions to avoid such systemic risks [16].

Together, these critiques suggest the need for rigorous oversight to ensure that PBD achieves its intended safety outcomes and is not undermined, whether by cost-cutting, misuse, or error.

### Disasters and Economic Incentives

Recent highly publicized fire events, such as the Grenfell fire (London, UK, 2017) and the Lacrosse fire (Melbourne, Australia, 2014) exposed the significant shortcomings in building codes and enforcement mechanisms highlighted above in Sec. 1.1. In particular, analyses of these disasters showed that building regulations lag behind modern construction practices [1, 4], oversight is fragmented [3], enforcement of the law is weak or confusing [4], and responsibilities (including liabilities) are unclear [57].^1^ In fact, the systemic vulnerabilities revealed by such disasters had already been recognized in the literature (e.g. [15, 16, 19]). These events can therefore be understood not as revealing entirely new problems, but rather as events of sufficient scale to bring these longstanding regulatory weaknesses to political and public attention.

Moreover, the two events underscore the critical role of economic incentives (i.e., financial drivers) for fire safety. In both cases, cost-cutting measures, coupled with fragmented regulatory oversight and weak enforcement, created catastrophic safety failures. Multiple authors have highlighted that developers and contractors frequently prioritize cost savings over safety (e.g., [6, 14, 57]), noting that this is reinforced by deregulation, privatized inspections, and the financialization of housing markets. This is enabled by legal mechanisms such as corporate phoenixing (i.e., the practice where directors liquidate a company to avoid debts and restart under a new name with the same assets/operations), which shield developers from accountability.

Regulatory reforms sparked by the two disasters appear to confirm that reactionary policy shifts often lead to overly prescriptive measures that increase costs without proportionate safety gains. For example, the UK’s ban on combustible cladding for buildings over 18 m was driven more by public outrage than by systematic cost–benefit analysis (hereafter, “CBA”), imposing significant retrofitting costs without tackling underlying systemic flaws [56]. Oswald et al. [57] extend this critique, linking Grenfell to broader market-driven pressures where deregulation and cost prioritization erode safety standards across the industry. Similarly, the reforms in Victoria after the Lacrosse fire risked creating compliance confusion—especially for small builders and property owners with limited resources—due to overly complex regulations^2^ [4]. Such confusion may drive up costs without proportional safety improvements [4].

These examples underscore a recurring theme: regulatory responses to fire disasters often oscillate between leniency and over-correction, rarely achieving an optimal balance of safety and economic efficiency. They also reveal a deeper tension: while legal frameworks are designed to enforce safety, economic incentives may not only encourage non-compliance but also drive actors to actively seek out loopholes and exploit regulatory grey areas.

### Scope

Sections 1.1 and 1.2 demonstrated that fire safety is intrinsically linked to legal and economic considerations. This aligns with prior work indicating that fire safety is not purely a technical issue (e.g. [3, 5, 11, 12, 21–23, 55]). This paper explores this interplay by linking fire safety with the discipline of “Law and Economics” (hereafter, “L&E”), i.e., the discipline focused on the economic analysis of the law (such as legal frameworks around fire safety). In particular, this article aims to: (i) introduce L&E as a research methodology and clarify its relevance for fire safety; (ii) explore how L&E principles can enhance legal frameworks around fire safety, particularly in clarifying incentive design, CBA, and risk allocation; (iii) examine how fire safety requirements and liability can be optimally specified via CBA.

Section 2 provides an introduction to L&E and its relationship with fire safety. Next, Sect. 3 develops a general cost model to evaluate expected outcomes of legal frameworks around fire safety. The model is applied in Sect. 4 to (a) compare prescriptive and PBD rules, and (b) assess the impact of liability rules on fire safety outcomes. Finally, conclusions are drawn in Sect. 5.

## Law and Economics and its Relationship with Fire Safety

### The Need for Law and Economics

**Fig. 1:**
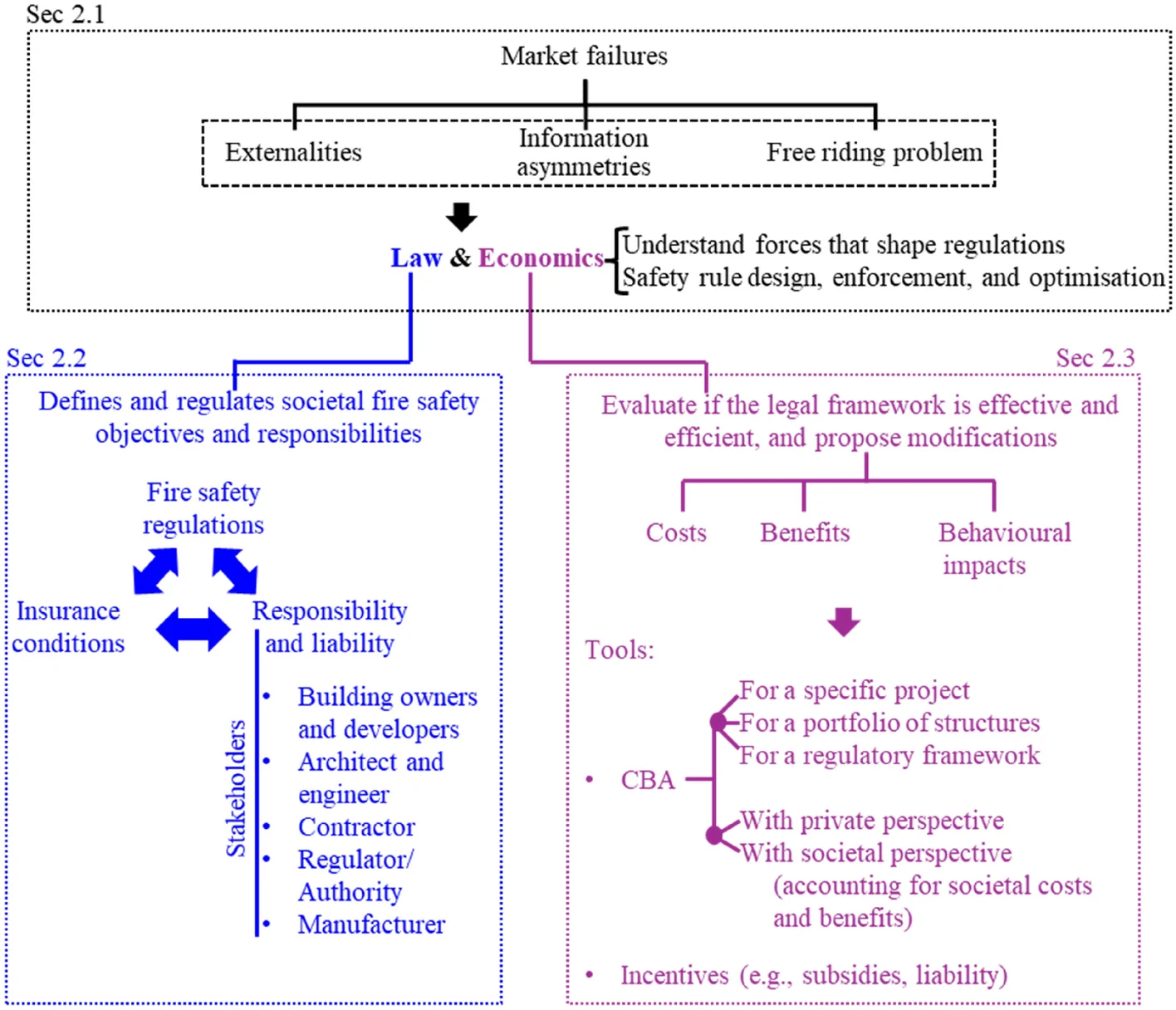
The role of law and economics in fire safety

L&E, as an interdisciplinary subdiscipline of both the legal and the economic disciplines, provides a framework for understanding the forces that shape fire safety outcomes and for identifying how safety rules can be designed, enforced, and optimized.^3^ In this context, legal frameworks specify safety requirements, enforcement mechanisms, and liability rules, while economics offers tools to assess the cost-effectiveness of safety measures and predict behavioral responses to incentives (e.g., [25]). Economics also explains why regulation is needed in the first place. In a perfectly efficient market, rational actors would naturally invest in the optimal level of fire safety to protect their private interests and those private interests would align with societal welfare, so that legal intervention would be unnecessary (and itself costly and therefore to be avoided, e.g., [26]). But in reality, several types of market failures (externalities, information asymmetries, and free riding problems) could prevent this from happening, thereby justifying the use of legal mechanisms to enhance societal welfare (e.g., [26, 27]).^4^ These concepts are elaborated in the following, as outlined in Fig. 1.

#### Externalities

The actions of one actor can impose costs on others, such as pollution, nuisance, or increased risk. When the actor does not bear these costs, a market failure arises in the form of (negative) externalities [30]. Because the actor faces only private costs rather than the full social costs, the activity is underpriced and therefore undertaken too frequently [30]. In the context of fire safety, externalities arise when fire risks extend beyond a single property (e.g., [18]), affecting neighboring properties and posing broader societal risks, including loss of life, environmental damage, and strain on public resources such as fire departments and hospitals. Without regulation, builders might underinvest in safety, knowing that some of the costs of a fire will be borne by others (e.g., the public fire department, neighboring properties). As demonstrated in the literature (e.g., [27, 31]), legal rules can internalize these external costs (i.e., ensure that costs are borne by the actors that create them), forcing actors to account for the broader societal impacts of their decisions.

#### Information Asymmetries

Due to the technical complexity of fire safety engineering, property owners, occupants, and regulators often lack the expertise to evaluate safety measures effectively. As a result of this information asymmetry, builders might cut corners to reduce costs without the end user realizing the increased risk.^5^ Legal rules can help to correct this market failure by setting minimum standards or mandatory information disclosures, while economics can determine how stringent those standards should be to avoid excessive burdens [18, 30]. To illustrate the concept, consider that current regulations often do not address a building’s repairability or structural stability after a fire. A business owner may not realize that a fire in their multi-story office building could render the entire structure unusable, potentially leading to complete operational shutdown. To mitigate such risks, a regulator could consider mandatory disclosure requirements that compel developers to provide clear and accessible data on the post-fire repairability of their buildings, allowing for more informed decision-making for intended occupants/users.

#### Public Goods and Free Riding Problem

Fire safety is a public good because it benefits society broadly.^6^ For example, a high level of fire safety in an individual building lowers the risk to adjacent properties. Individual actors may, however, underinvest as it is not always privately profitable to install safety measures up to a high standard. Besides that, individual actors may expect to be able to take advantage of the investments of others (i.e., free riding). Therefore, without public intervention, the public good of fire safety is likely to be underprovided. In this context, Kunreuther [32] provides a valuable parallel from disaster mitigation after Hurricane Katrina, describing a “natural disaster syndrome” where individuals and firms underinvest in preventive measures due to underestimated risks and financial constraints, relying instead on insurance or government aid. Applied to fire safety, builders may similarly forgo costly upgrades (e.g., fire-resistant materials), shifting the burden to insurers or taxpayers. To correct these misaligned incentives, Kunreuther [32] advocates for public–private partnerships and using tools such as insurance discounts (i.e., premium adjustments tied to safety upgrades) or subsidies that promote proactive safety investments. This approach reduces dependence on post-disaster aid and leverages market mechanisms to reinforce societally beneficial behaviors (here: private investment in fire safety), thus aligning private risk management decisions with public safety goals. In a similar vein, Spinardi et al. [2] and Swan [33] note that the socioeconomic disparities leave disadvantaged communities reliant on public intervention, underscoring the need for comprehensive strategies that integrate insurance, regulation, and public investment in fire safety infrastructure.

#### Solutions

These market failures highlight that a purely market-driven approach to fire safety is insufficient. Legal rules can correct externalities by imposing liability, reduce information asymmetries through standards and transparency, and ensure the provision of public goods via minimum requirements. Economic analysis complements this by offering tools to evaluate the cost-effectiveness of regulations and predict behavioral responses to incentives [30]. Viscusi [34] highlights the importance of balancing risk reduction against opportunity costs (i.e., recognizing that resources spent on increasing safety cannot be spent on other endeavors), and argues that overly strict regulations can lead to diminishing returns^7^ while lenient ones can incentivize dangerous cost-cutting practices. Similarly, Kunreuther & Heal [37] note that individual actors tend to underinvest in safety unless regulatory or market-based mechanisms align incentives.

Cognitive biases can also distort risk perception, which further reinforces the case for incorporating L&E considerations when developing fire safety policies. For example, Kinsey et al. [38] identify a range of heuristics (such as availability bias, probability neglect, anchoring, default bias, optimism bias, and familiarity bias) that may impair general fire engineering practice, including code interpretation and risk modelling. Pacces & Visscher [30] and Korobkin & Ulen [39] suggest that such cognitive biases necessitate regulations that account for irrational decision-making. Integrating an L&E perspective into the design of fire safety regulation can therefore enhance both safety outcomes and economic efficiency. Indeed, L&E provides the analytical tools to design legal rules that align individual incentives with societal welfare, thereby addressing market failures (i.e., internalizing externalities, correcting information asymmetries, and ensuring the provision of public goods) and promoting efficient outcomes [26, 27]. Here, “efficiency” refers to the optimal allocation of resources to maximize societal welfare.^8^ In the context of fire safety, this translates into regulations that achieve the highest level of safety at the lowest possible cost, both for society as a whole. Efficiency also requires optimal enforcement strategies that balance sanctions and monitoring costs to maximize compliance without overburdening stakeholders (e.g., [18]). In the following, Sec. 2.2 and 2.3 dig deeper into the role of the legal framework and the role of economics in the attainment of fire safety in the built environment.

### The Role of the Legal Framework

The legal framework defines and regulates societal fire safety objectives and responsibilities by (i) establishing fire safety requirements, which broadly refer to the rules that (implicitly) define adequate levels of fire safety in buildings; (ii) specifying enforcement mechanisms and allocating responsibility. Legal frameworks vary across jurisdictions^9^ and encompass both prescriptive and PBD approaches [43, 55]. The requirements and enforcement mechanisms are in the following jointly referred to as “(fire safety) regulation.” In case of failure/breaches of fire safety regulation, liability rules help answer questions of responsibility, by dictating who is accountable for ensuring compliance and who bears the consequences when failures (and resulting damages) occur. These liability rules also differ across jurisdictions and can trigger civil liability aimed at compensation, criminal liability intended to punish, incapacitate and/or deter, or both. Finally, the level of fire safety achieved in buildings cannot be seen separate from a third aspect: insurance considerations. In some jurisdictions, insurance considerations can also be placed under the umbrella of fire safety regulation, in the form of mandatory insurance schemes.^10^

#### Fire Safety Regulation

What constitutes a fire safety requirement varies depending on jurisdiction and regulatory philosophy. In Belgium (Royal Decree of 7 July 1994 [44]), fire safety regulations are highly prescriptive, specifying detailed technical criteria such as minimum fire resistance ratings, compartmentation rules, and evacuation facilities. In contrast, the UK's Building Regulations provide a more PBD approach, defining fire safety objectives rather than rigid design solutions (e.g., [8]). Such a distinction also affects legal accountability:

- In a prescriptive system (e.g., Belgium), compliance is primarily a matter of following explicit technical standards, making liability more straightforward: if a design meets the code, legal responsibility is typically limited. However, this does not necessarily fully shield actors from liability. Negligence may still be established if, for example, the chosen solution – although technically compliant – was foreseeably inadequate given the specific context, or if the actor failed to account for risks not addressed by the technical standards (e.g., the cooling phase failure of structural members is a well documented phenomenon which is not consistently addressed by standard fire-resistance ratings; [45, 46]). Courts may also find negligence where there is a lack of due diligence, failure to update designs in light of new knowledge, or disregard for best practices that go beyond the minimum legal standards. Wright [47] emphasizes that professional negligence requires engineers to meet the standard of an ordinary skilled professional, meaning that compliance with prescriptive codes may not suffice if new risks, such as those posed by modern materials, are foreseeable.
- In a PBD system (e.g., UK^11^), liability is more complex, as architects and engineers must prove that their solutions achieve adequate safety. This increases the potential for disputes in case of failure and shifts responsibility from regulators to professionals [47]. Spinardi [55] notes that the shift to PBD increases engineers’ exposure to liability due to the need to justify their designs against probabilistic fire risks, which are inherently uncertain and difficult to quantify. On the other hand, Meacham et al. [43] note that globally, PBD building regulations face challenges in defining measurable safety criteria and ensuring accountability, particularly as societal expectations evolve with climate change and urbanization. The flexibility of PBD can drive cost-effective innovation in fire safety, but it also risks inconsistent enforcement, underscoring the need for robust legal frameworks to support PBD’s economic benefits [5, 48].
- The disparate legal status (legally binding vs advisory) of fire safety requirements further complicates legal accountability. Some requirements function as mandatory laws, enforceable with penalties for non-compliance (e.g., in the UK, ignoring the relevant requirements of the building regulations is a criminal offence and may lead to a conviction, an unlimited fine and imprisonment; [49]), while others act as guidance documents that offer best practices but allow for deviations if equivalent safety can be demonstrated (e.g., again for the UK, Approved Document B, is non-binding and allows deviations [49]).

Fire safety regulations worldwide are enforced through various methods, including regulatory approvals, (surprise) inspections, audits, penalties, cognitive infrastructure (e.g., where active and passive fire safety measures in buildings are monitored continuously using automated sensors) and private enforcement via civil lawsuits (e.g., [50] and [1]). In the UK, inspections paired with substantial (criminal) penalties deter non-compliance, while Belgium uses administrative tools like permit denials, which are cost-effective but less punitive. These enforcement strategies reflect a trade-off between safety and economic efficiency, with strict approaches ensuring compliance at the cost of higher regulatory burdens for stakeholders, and lighter approaches potentially compromising safety.

#### Legal Responsibility and Liability

Legal responsibility in fire safety varies across jurisdictions and depends on the type of legal framework in place. It is important to distinguish between civil liability, which is primarily aimed at compensating victims for losses suffered, and criminal liability, which is aimed at punishment, incapacitation, and deterrence, and applies where conduct is sufficiently culpable [47, 51]. The two can overlap: the same fire event may simultaneously give rise to civil claims by affected parties and criminal prosecution by public authorities [26, 27]. The standard of proof and the consequences differ substantially: criminal liability requires proof beyond reasonable doubt and may result in imprisonment or fines, while civil liability requires causality between the harm and the damage and the probability that the harm can be attributed to the stakeholder and results in damages awards [27, 52]. Generally, multiple stakeholders can be held responsible for failures and resulting damages [47]. Their roles are summarized in Table 1.

**Table 1:** Liability fire safety stakeholders

Stakeholder	Liability	Example
Building Owners and Developers	Often bear primary legal responsibility for ensuring that fire safety measures comply with fire safety regulations. If a fire occurs due to negligence or non-compliance, they may face fines, lawsuits, or criminal charges	In the UK, the Regulatory Reform (Fire Safety) Order 2005 mandates owners to mitigate risks proactively [3]. In the U.S., owners of vacant properties face fines up to $1,000/day for fire code violations [5]
Architects and Engineers	Responsible for ensuring that their fire safety strategies achieve adequate protection. If a fire safety design fails in practice, liability (in the form of professional negligence) may fall on them. In PBD systems, they face increased scrutiny, as they must demonstrate that possibly innovative designs meet safety objectives, increasing their exposure to legal risks [53–55] argue that the lack of a standardized accreditation system for fire safety engineers exacerbates these risks, as insufficient competency levels can lead to design failures	After the Lacrosse fire, architects were sued for approving flammable cladding, despite arguing it met performance goals [14]
Contractors and Construction Firms	Responsible for properly implementing fire safety measures (i.e., executing designs faithfully, using approved materials). If they use substandard materials or ignore requirements in codes, they may be liable	The Grenfell Tower fire revealed failures related to the use of combustible cladding, placing legal responsibility on contractors as well [56, 57]. In the Lacrosse fire case, the applicable legal regime placed a guarantee obligation on the contractor, making the contractor liable without fault [58]
Regulators and Authorities	Government agencies play a supervisory role, ensuring that fire safety regulations are enforced and designs are approved. However, weak enforcement or unclear guidelines can lead to liability gaps, where no single party is fully accountable. Regulatory bodies will rarely be directly liable, but weak oversight invites criticism	Post-Grenfell, the local authority’s building control department of the Royal Borough of Kensington and Chelsea (London, England) was found by the Grenfell Tower Inquiry to have failed to adequately scrutinize the design and choice of materials during the refurbishment, and to have approved plans without site checks [3]
Manufacturers and Suppliers	If a fire disaster is caused by, for example, a defective construction product or faulty alarm system, liability may extend to the companies that supplied those products, under product liability rules	The use of non-compliant cladding materials in high-rise buildings, as seen in Australia, has led to significant financial and legal repercussions for suppliers and manufacturers [14] Nguyen et al. [59] emphasize the role of manufacturers in ensuring the fire performance of (façade) materials, noting that incidents like Grenfell highlight the need for stricter product testing and certification to prevent liability disputes

The multiple possible liability pathways listed in Table 1 illustrate the importance of legal clarity in assigning responsibilities for fire safety. If roles and liabilities are poorly defined, stakeholders may attempt to shift blame, as seen in the Grenfell Tower fire case, where contractors, architects, building owners, and regulators each deflected responsibility [60]. The complexity of modern construction projects, with multiple layers of subcontracting and fragmented oversight, exacerbates these challenges, making it often difficult to precisely pinpoint where responsibilities lie [57]. This fragmentation of accountability is not a new phenomenon: 15, 16) has shown that diffuse regulatory regimes (according to May, particularly those built around PBD) tend to erode clear lines of responsibility, as the flexibility afforded to stakeholders simultaneously obscures who is ultimately accountable when safety outcomes fall short. McEvoy [61] provides a historical parallel with the Triangle Shirtwaist Factory fire of 1911, where unclear accountability and inadequate regulations allowed employers to externalize safety costs, leading to catastrophic losses, a pattern May [16] identifies as endemic to regulatory regimes that lack robust accountability mechanisms.

A further structural dimension of liability concerns how responsibility is apportioned when multiple parties contribute to a fire safety failure, a situation that is, as Table 1 illustrates, the rule rather than the exception in construction. Two principal apportionment rules are recognized in the L&E literature (e.g. [51, 62]). Under joint and several liability, any one stakeholder can be held liable for the full extent of the harm, regardless of their individual share of fault, with the burden falling on defendants to seek contribution from one another. This approach protects victims, but may overdeter stakeholders with only marginal contributions to the harm [62]. Under proportionate liability, each stakeholder is liable only for the share of the damage attributable to their conduct. This reduces overdeterrence risks but may leave victims under-compensated where a stakeholder is insolvent or cannot be identified [51, 62]. From an L&E perspective, joint and several liability is generally favored where one party is better placed to monitor and control the behavior of others (e.g., a principal contractor overseeing subcontractors), while proportionate liability may be preferable where parties act independently and cannot control one another's conduct [51, 63]. It is also worth noting that liability exposure varies with the nature of the conduct: active negligent acts typically attract greater liability than omissions, though omissions can equally ground liability where a duty of care to act exists (e.g., reasonable care, as judged by professional standards, see also Sect. 4.2.3.; [27, 52]). Similarly, distinctions are drawn between ordinary negligence, gross negligence, and recklessness, with more culpable conduct typically exposing stakeholders to greater damages, and in some jurisdictions to punitive or exemplary damages beyond mere compensation [27, 52].

However, because the fire safety socio-technical system is complex, the effects of liability changes on the cost-effective delivery of fire safe buildings are not obvious [64, 65]. Thus, it is not recommendable for a legal system to assign responsibilities without first investigating how liabilities should be best allocated. The L&E doctrine clarifies the question of liability allocation through the concept of the “cheapest cost avoider”, introduced by Calabresi [66]: liability should be allocated to the party best positioned to prevent harm at the lowest cost, thereby promoting efficient risk reduction and minimizing overall societal costs. In the context of fire safety, this means assigning primary liability to, for example, manufacturers of building materials if they can most affordably ensure performance, or to engineers if their expertise enables them to foresee and avoid risks more effectively than other stakeholders (e.g., [63]).

It should be noted that the cheapest cost avoider principle operates at the level of primary liability allocation, i.e. identifying which party should bear responsibility in the first instance; and is therefore distinct from the question of apportionment among multiple liable parties discussed above. In practice, the two interact: where one party is identified as the cheapest cost avoider, this typically favors a proportionate rather than joint and several liability structure, since placing the full burden of harm on a party with only a marginal contribution to the risk would distort the incentives the principle seeks to align [51, 66].

#### Insurance Considerations

Insurance plays a critical role in risk-based decision-making, influencing both compliance and investment in safety measures. Insurance mechanisms can incentivize proactive (such as fire) safety investments by offering premium reductions for buildings that exceed minimum safety standards [32]. For example, to align private incentives with public safety goals (e.g. [34]), insurers may provide discounts for properties equipped with sprinkler systems or compartmentation. However, insurance markets also face challenges in accurately pricing fire risks, particularly in regions with high wildfire exposure or aging building stock [5]. Furthermore, the resolution of available fire statistics is limited, meaning that statistical pricing is difficult. For example, available fire statistics do not differentiate in function of the compartmentation classification [67].

From an L&E perspective, insurance mechanisms can complement regulatory enforcement by internalizing the externalities. Fire losses are distributed across policyholders while encouraging risk-reducing behaviors [31]. For example, Law [68] notes that insurers have increasingly excluded coverage for combustible cladding-related claims, creating market pressure for surveyors to exceed minimum compliance. On the other hand, where insurance premiums do not reasonably reduce with increases in fire protection, a net disincentive for private fire safety investments occurs.

While insurance mechanisms can help internalize externalities by pricing risk, they simultaneously introduce the risk of moral hazard,^12^ as insured parties may underinvest in safety if they perceive insurance as a substitute for preventive measures [32]. This differs from free-riding, where an actor (intentionally) benefits from safety investments by others without contributing to the cost. Effectively, free-riding is a failure of contribution, while moral hazard is a failure of care induced by the presence of insurance. Often, insurers mitigate this effect by requiring compliance with regulations as a condition of coverage, reinforcing the role of legal frameworks in shaping risk management practices [34].

### The Role of Economics

While law defines obligations and accountability, economics helps evaluate whether the legal framework is effective and efficient by analyzing its costs, benefits, and behavioral impacts. Van den Bergh & Visscher [18] stress that enforcement strategies must be economically optimized, balancing deterrence with cost-efficiency. This is particularly pertinent in fire safety, where inspections are infrequent and violations can go undetected until a fire occurs. In such settings, precautionary strategies such as requiring fire certification before occupancy may be more effective than post-incident penalties. In the context of licensing for rental properties, Samuel et al. [70] provide a calibrated economic model which shows that licensing and rental property compliance schemes (which typically include fire safety provisions) improve safety, but also raise rents and reduce the supply of low-cost housing. This highlights a critical trade-off in the broader context of (fire) safety regulation: overly stringent or poorly calibrated requirements may reduce building affordability, resulting in a net disbenefit to the most vulnerable groups in society [35]. Fire safety policies must therefore balance cost, feasibility, and public safety, making economic analysis a crucial tool for regulators and engineers. Economic tools relevant to this process are reviewed in the next subsections.

#### Cost–Benefit Analysis

Fire safety measures come with costs, such as sprinkler installation and maintenance, and CBA evaluates whether the resulting safety benefits justify these expenses. Such analysis can be done at the level of a project, but also at the level of the building stock (e.g., all multi-residency buildings in a jurisdiction), guiding policymakers and engineers in balancing trade-offs between investments and economic feasibility [8, 23, 41]. For example, Van Coile et al. [41] apply the Present Net Value approach to a warehouse case study, showing that optimal compartmentation yields higher net benefits than both less stringent safety schemes with limited compartmentation and more stringent schemes that combine sprinklers with high levels of compartmentation. Their analysis illustrates that well-designed economic models can guide efficient safety design, provided they account for both direct and indirect costs and the value of risk reduction. Performing a CBA in order to guide the development of regulations is considered best practice and is often mandatory [35]. Viscusi [34] further argues that safety measures must be evaluated not just for their direct benefits (i.e., reductions in expected fire-induced losses) but for their broader economic impacts.

A third level for the CBA entails evaluating costs and benefits not for a specific project or for a proposed requirement for the entire building stock (e.g., banning combustible cladding), but on the level of the legal framework itself. This allows to answer questions on how responsibilities are most efficiently allocated, or whether a mandatory certification scheme for engineers is expected to result in a net benefit to society. While CBA has been applied to specific fire safety measures and regulatory requirements (including sprinkler mandates and building stock-level interventions, e.g., [36, 71, 72]), the application of CBA to the design of the legal framework itself, remains limited. However, such investigations also present challenges. As Nardinelli [73] demonstrates, CBA often suffers from errors in defining baselines or failing to account for the heterogeneous distribution of risks and benefits across different social groups. Ogus [74] highlights the difficulty of assessing counterfactuals (e.g., what would happen without regulation) and of predicting changes in compliance levels (e.g., to what extent building owners will adhere to fire codes, as a function of the liability regime). This is further complicated by incomplete data on current fire incidents and compliance rates [33],empirical estimates of the total societal cost of fire (decomposed into costs of anticipation, response, and consequences) remain scarce and context-specific [23, 75]. While many countries maintain annual fire-incident records, these datasets may under-represent rare but catastrophic events unless they cover a sufficiently long time span and a large enough sample of buildings. Alternatively, modelling can be used to compensate for a lack of data (as in [76]).

When performing CBA, it is crucial to distinguish between analyses with a private perspective and those with a societal perspective [23, 41, 77]. In private CBA, the decision maker can value costs and benefits as they see fit. Societal losses such as pollutant releases into the environment are, absent a legal mechanism internalizing them, generally considered of no monetary value to the private decision maker [78, 79]. Societal CBA, on the other hand, must include all public impacts. Meacham [5] refers to the economic ripple effects of fire disasters on communities, such as lost tax revenue or increased healthcare costs. The difference between the private and the societal optimum then creates a point of tension, whereby the private decision maker is inclined to over- or underinvest in safety relative to the societal optimum. This is explored further in Sect. 4.2 when discussing liability regimes. In order to optimize the legal framework, it is thus necessary to consider both perspectives: (i) identify the societally preferred solution, and (ii) be cognizant of the behavior of the private decision makers. This evaluation may show that there is a need to introduce incentives (e.g., liability, subsidies, reputational damage and market mechanisms) to influence the private decision maker’s preferences.

#### Incentives

The legal framework influences how businesses and individuals behave. In this regard, the rational choice theory within L&E [18] provides a useful – though theoretical^13^ – lens for understanding how stakeholders respond to the law. According to this theory, individuals and organizations make decisions based on a rational assessment of costs and benefits. For example, stronger penalties can deter non-compliance, while financial incentives (e.g., insurance discounts for safer buildings) can encourage proactive fire safety investments. Hirshleifer [80] explores in detail how penalties and rewards influence compliance, noting that well-calibrated financial penalties can deter unsafe practices, while tax incentives or subsidies can encourage proactive safety investments. However, Viscusi [81] cautions that fire safety regulations, such as flammability standards for carpets, can also yield unintended economic consequences if behavioral responses, such as reduced vigilance, are ignored. His analysis of the US Consumer Product Safety Act [82] reveals that safety benefits may be overstated without accounting for such offsetting effects, urging a more dynamic cost–benefit approach that integrates human behavior into economic models. This suggests that poorly designed incentives can also be counterproductive. Thus, a careful evaluation of the foreseeable effects of an incentive scheme should be done prior to implementation.

Overall, by leveraging L&E approaches, the legal framework for fire safety can be shaped through necessary prior evaluations, resulting in a framework that is more effective, adaptable, and economically sound, while accounting for both safety outcomes and economic trade-offs. Building on these insights, the following section develops a simple economic model which allows to evaluate the expected outcomes of legal frameworks for fire safety, and thus compare the expected impact of alternative approaches.

## Economic Model For Fire Safety Legal Frameworks

A formal economic model is developed in the following to compare alternatives related to legal frameworks around fire safety (e.g., different liability schemes), hereafter, “regulatory approaches”, and to formally identify (in)efficiencies induced by a considered regulatory approach. This differs from a Regulatory Impact Assessment (hereafter, “RIA”), which is a tool sometimes used by governments to evaluate the (societal) costs and benefits of specific proposed regulatory measures or requirements [83]. Instead, our model does not assess a specific regulatory proposal, but rather the structural design of legal frameworks around fire safety itself, which is a level of abstraction that goes beyond what standard RIA practice [83] addresses.^14^ The model builds on standard CBA frameworks (e.g., [41]) and applies to both societal and private evaluations. It is intentionally kept simple to introduce the concept of L&E evaluations for fire safety. When considering a private perspective, only a single actor making decisions is considered (i.e., builder or fire safety engineer). Situations involving competing responsibilities among other private stakeholders (e.g., architects, fire engineers, building owners) are not considered.

A general CBA formulation evaluates the net lifetime utility *Y* of an action as *Y=B-C-D*, where *B* is the benefit derived from the action; *C* is the cost of implementation; *D* represents the direct and indirect losses resulting from the action. The valuation of these variables for a given situation depends on the perspective of the analysis, as highlighted in Sect. 2.3.1.

In the following, we investigate decision problems whereby the decision parameter can be broadly mapped to the cost of implementation *C*, and where the benefit derived from the decision is a reduction in the losses *D*. The problem is then simplified to minimizing the total cost *Z*, i.e., Eq. (1). In other words, we consider that the benefit *B* is independent of the decision parameter. This means that, for example, the benefit derived from the use of a building is considered independent of the fire safety investment. The optimal cost of implementation (i.e., optimal investment level) is where the marginal cost of additional safety investment equals the marginal reduction in expected damages, as mathematically specified by Eq. (2). An evaluation from a societal perspective then results in the societally optimum investment level, while an evaluation from a private perspective gives the optimum investment level for the private decision maker.

1 *Z=C+D*

2 $$\frac{dZ}{dC}=1+\frac{dD}{dC}=0 \Rightarrow \frac{dD}{dC}=-1$$

The fundamental principle of Eq. (2) underpins the analysis of regulatory approaches around fire safety in the following sections. Section 4.1 explores how prescriptive and PBD frameworks result in differing levels of investment and incentives for innovation, and how enforcement costs impact the optimum design rule. Next, Sect. 4.2 examines how liability regimes influence decision-makers' incentives for fire safety investment. Three regimes are compared: (i) strict liability, (ii) negligence based on code-compliance, and (iii) negligence based on due-diligence. The analyses are simplified with a view to gaining insights. The goal is thus not to present an accurate prediction of how decision makers will act in specific circumstances. The rational choice theory is adopted, fully recognizing its limitations (see 2.3.2). The model considers a single objective, and thus does not take account of the interaction between possibly competing societal objectives such as fire safety and energy efficiency considerations. For multi-objective optimization in the context of fire safety engineering, reference is made to Chaudhary, Gernay & Van Coile [85].

## Model Applications

### Prescriptive Rules vs. PBD Rules

The differences in economic efficiency between prescriptive and PBD regulatory approaches can be conceptually demonstrated with the model outlined in Sect. 3. In this analysis, we consider the PBD rule to require investment “as far as reasonably practicable” to evaluate what can be achieved with such an approach. As elaborated elsewhere [86, 87], such a requirement is to be understood as requiring investment in safety as far as economically feasible from a societal perspective. Alternative PBD functional requirements can be considered, and can result in different conclusions (e.g., where a PBD rule would be operationalized by a functional requirement of “no repair needed to the structure after fire”). For simplicity, discussions on discounting are omitted.

#### Total Cost of Fire and Deadweight Loss

Consider a decision problem on the appropriate level of fire safety investment (e.g., sprinklers or fireproofing) with total direct cost *C≥ 0*. The expected loss from fire incidents *D* is a function of the investment made, i.e., *D(C)*, and accounts for both the frequency of fire occurrence and the severity of damage, which should be interpreted broadly to include the full spectrum of fire-related losses [41, 67]. *D* decreases with increased safety investment *C*. For illustrative purposes, an inverse-hyperbolic function is adopted—see Eq. (3), where $\frac{k}{a}>0$ is the expected fire-induced loss without the safety investment. Here, *k > 0* and *a > 0* are positive constants for a given building. In practice, both parameters will vary across buildings: *k > 0* reflects the potential loss magnitude, and will be higher for e.g. buildings with greater occupancy, higher fuel loads, or more severe consequences of failure; and *a>0* captures inherent safety (e.g., recognizing that – for example – the non-fire design of the building structure will already result in some level of resources spent in a manner which is beneficial with respect to performance in fire, and that for *C* approaching zero the expected fire losses do not tend to infinity).

3 $$D\left(C\right)=\frac{k}{C+a}$$

Considering Eq. (3), the damage magnitude decreases with increasing *C* and is convex, i.e., $D{\prime}(C)=-\frac{k}{{\left(C+a\right)}^{2}}<0$ and ${D}^{{\prime}{\prime}}\left(C\right)= \frac{2k}{{\left(C+a\right)}^{3}}>0 ,$ capturing diminishing returns with increasing investment. Diminishing returns is a common feature of safety investments [23], where initial investments significantly reduce the expected loss, but additional investments yield progressively smaller reductions, see for example the reductions in failure probability for steel beams with increasing insulation thickness in [88].

From a societal perspective, both the investment *C* and the expected fire-induced losses are relevant cost components. They both constitute an economic loss to society (i.e., monetary resources which are not available for increasing societal welfare in another way). The total expected cost for society is thus specified by Eq. (4).

4 $$Z\left(C\right)=C+\frac{k}{C+a}$$

The first-order condition for minimizing this expression is given by Eq. (5), which defines the societally optimal investment level, denoted as ${C}^{*}.$ This solution is feasible only if the marginal benefit at zero investment exceeds the marginal cost, i.e., if $\sqrt{k}>a$. Otherwise, the optimum occurs at the corner solution ${C}^{*}=0.$ The minimized total cost at ${C}^{*}$ is given by Eq. (6), and is further denoted as ${Z}^{*}$.

5 $$\frac{dZ}{dC}=1-\frac{k}{{\left({C}^{ }+a\right)}^{2}}=0\Rightarrow {C}^{*}=\sqrt{k}-a$$

6 $$Z\left({C}^{*}\right)={C}^{*}+ \frac{k}{{C}^{*}+a} =2\sqrt{k}-a={Z}^{*}$$

It is now possible to evaluate whether the investment level chosen by a private decision maker under a PBD rule or prescriptive rule aligns with the societally optimum investment. The evaluation is done first for a private decision-maker under a PBD rule. The PBD rule stipulates that investment is required as far as economically feasible. Under caveats elaborated elsewhere [86, 87], i.e., tolerability, this entails a cost optimization. For simplicity, the analysis assumes an environment where the private decision-maker fully internalizes all costs (i.e., both *C* and *D* in this example). In other words, the decision maker not only fully carries the investment cost but also all other expected losses resulting from fire incidents. This is a simplifying assumption since externalities are generally not fully internalized, meaning that the decision-maker often does not carry the full cost of fire because of insurance, limited liability, or regulatory gaps. It is further assumed that there are no additional cost components for the private decision maker (e.g., sentimental or aesthetic costs). Under the listed assumptions, the expected fire-induced losses are described by the same model of Eq. (3). The total expected cost function that the private decision-maker minimizes is thus given by Eq. (4), and the minimization of this total cost follows Eqs.(5) and (6). The minimum total cost of fire is ${Z}_{PBD}=2\sqrt{k}-a$, and is obtained for the investment level ${C}_{PBD}=\sqrt{k}-a$. Considering the assumptions listed above, the total cost ${Z}_{PBD}$ is both the total cost for the private decision-maker, as well as for society. Thus, no distinction in notation is introduced here.

Under a prescriptive rule, the regulator mandates a specific investment level, i.e., $C = {C}_{pr}$. This yields the total cost of Eq. (7). A distinction can be made between (i) strict prescription, where the prescribed investment has to be adopted, and (ii) minimum prescription, where the private decision maker is allowed to exceed the prescribed investment level. In case of strict prescription, Eq. (7) directly applies, i.e., ${Z}_{pr}^{str}={Z}_{pr}$. In case of minimum prescription, the private decision-maker will invest beyond ${C}_{pr}$ when the private optimum investment level is higher. In other words, the private decision maker will then invest up to ${C}_{PBD}$, resulting in ${Z}_{pr}^{min}={Z}_{PBD}$. Under the assumptions listed above (externalities are internalized, no additional private cost components), this total cost again applies both to the societal and the private perspectives.

7 $${Z}_{pr} ={C}_{pr} +\frac{ k}{{C}_{pr} +a}$$

If the design rule results in a difference between the investment level adopted by the private decision-maker and the societally optimum investment level, then a deadweight loss (DWL) arises. The DWL is the loss of economic efficiency resulting from a suboptimal allocation of societal resources and is thus evaluated from a societal perspective. It is a measure of the loss of welfare to society resulting from the inefficiency in the design rule. A private equivalent can be construed (i.e., the private cost incurred when a rule requires a private decision-maker to deviate from their optimum) and can be referred to as “private efficiency loss”.

Given the assumptions in Sec. 4.1.1, ${C}_{PBD}={C}^{*}$, and thus no DWL is incurred under the PBD rule. As societal and private costs map perfectly (externalities internalized, no additional private costs), there is also no private loss of efficiency. Under the strict prescriptive rule, a DWL is incurred when ${C}_{pr}\ne {C}^{*}$, i.e., Eq. (8). Note that ${Z}_{pr}$ ≥ ${Z}^{*}$, since ${Z}^{*}$ is the minimum societal cost. In case of a minimum prescriptive rule, a DWL is incurred and Eq. (8) applies when ${C}_{pr}>{C}^{*}$, while no DWL is incurred when ${C}_{pr}<{C}^{*}$ since the private decision-maker will then (on their own accord) invest beyond ${C}_{pr}$ up to ${C}^{*}$.

8 $$DWL={Z}_{pr}-{Z}^{*}$$

**Fig. 2:**
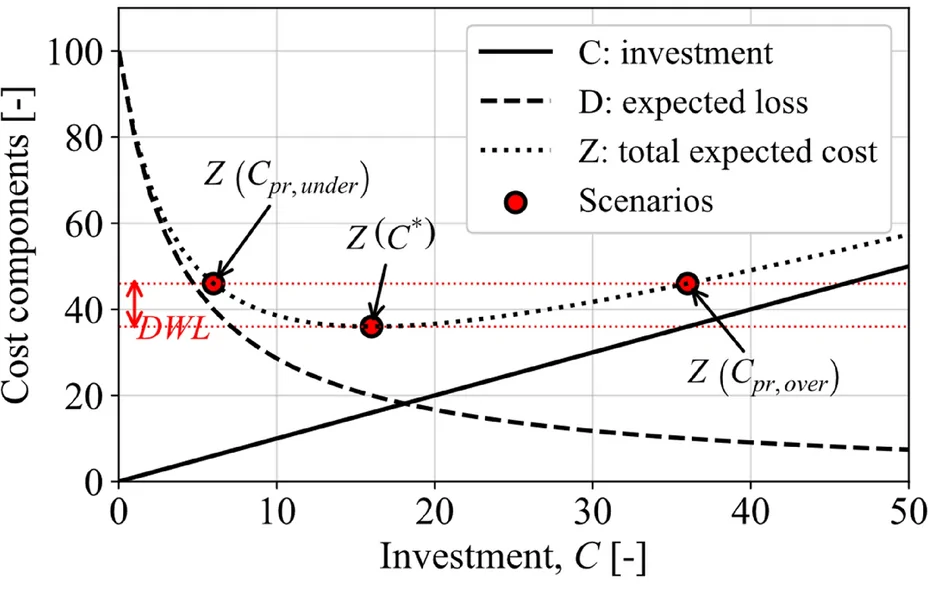
Investment cost, fire-induced losses and total cost of fire as a function of the investment cost, for $\frac{k}{a}=100$ and *a = 4*. Scenario data from Table 2 included, with indication of DWL

The DWL quantification above demonstrates an inherent inefficiency in prescriptive rules, i.e., a societal cost of fire in excess of what can be achieved with alternative rules. To illustrate these concepts numerically, consider an evaluation with scaled hypothetical parameter values $\frac{k}{a}=100$, *a = 4* (and thus *k = 400*). Considering these parameter values and Eqs.(3) and (4), Fig. 2 visualizes the total cost of fire as a function of the investment level *C*. Due to the scaling of the parameters, $Z\left(0\right)=100$, represents the total cost of fire in absence of any dedicated fire safety investment. The societal optimal investment ${C}^{*}=\sqrt{400}-4=16$, resulting in the minimum value for the societal total cost of fire ${Z}^{*}=2\sqrt{400}-4=36$. Two scenarios are considered regarding prescriptive requirements: a scenario ${C}_{pr}={C}_{pr,under}=6$ (under-investment) and a scenario ${{C}_{pr}=C}_{pr,over}=36$ (over-investment). The resulting total cost and deadweight losses are summarized in Table 2 for the different design rules considered. Since this analysis assumes that all externalities are internalized and there are no additional private costs, the conclusions regarding DWL apply directly to private efficiency loss as well. Figure 2 visualizes the same data.

Table 2 for the different design rules considered. Since this analysis assumes that all externalities are internalized and there are no additional private costs, the conclusions regarding DWL apply directly to private efficiency loss as well. Figure 2 visualizes the same data.

**Table 2:** Overview of the privately chosen investment level, total cost of fire and deadweight loss, considering different design rules and prescriptive requirements

Rule | Scenario	Investment, total cost, and deadweight loss	
PBD rule	${C}_{PBD}={C}^{*}=16$ ${Z}_{PBD}= {Z}^{*}= 36$ $DWL={Z}_{PBD}-{Z}^{*}=0$	
Prescriptive investment	${C}_{pr}={C}_{pr,under}=6$	${C}_{pr}={C}_{pr,over}=36$
Strict prescriptive rule	${C}_{pr}^{str}={C}_{pr}=6$ ${Z}_{pr}^{str}={Z}_{pr}=46$ $DWL={Z}_{pr}^{str}-{Z}^{*}=10$	${C}_{pr}^{str}={C}_{pr}=36$ ${Z}_{pr}^{str}={Z}_{pr}=46$ $DWL={Z}_{pr}^{str}-{Z}^{*}=10$
Minimum prescriptive rule	${C}_{pr}^{min}={C}^{*}=16$ ${Z}_{pr}^{min}={Z}_{PBD}=36$ $DWL={Z}_{pr}^{min}-{Z}^{*}=0$	${C}_{pr}^{min}={C}_{pr}=36$ ${Z}_{pr}^{min}={Z}_{pr}=46$ $DWL={Z}_{pr}^{min}-{Z}^{*}=10$

Table 2 indicates that under a minimum prescriptive rule, there is no DWL when the prescriptive investment level is below the optimum, since the internalization of external costs incentivizes the private decision maker to exceed the prescriptive investment level ${C}_{pr}$ and invest up to ${C}^{*}$. As is also the case for the PBD rule, this incentive diminishes if only a fraction of the costs are internalized, e.g., due to (lack of) liability assignment or insurance shifting damages to others, leading to potential under-investment at or near ${C}_{pr}$. This highlights the importance of liability and regulatory design in aligning private and societal optima, as discussed further in Sect. 4.2. One could argue that DWL can also be avoided by an appropriate specification of ${C}_{pr}$. However, even if ${C}_{pr}$ can be well aligned with ${C}^{*}$ for a specific situation, it is unlikely to be well-suited across buildings since individual buildings vary significantly in their characteristics (which implies varying *k* and *a*). This result is important as it indicates that the often-proclaimed inefficiency of prescriptive rules directly results from its mode of operation and cannot be fully addressed by improving the prescriptions themselves. Since the model and assumptions underlying this result are simplified, it may still be possible to specify specific situations where prescriptive rules are efficient (e.g., by taking into account enforcement costs, see 4.1.3 below). For completeness, it should further be noted that the analysis above considers a static decision context and does not account for changes over the building's lifetime (e.g., maintenance, change of use) that may shift the effective values of *k* and *a*.

#### Innovation Incentives

PBD rules can encourage fire safety engineering innovation by allowing decision-makers to optimize not only the baseline level of investment *C,* but also the methods by which safety is achieved. Suppose the decision-maker can exert an innovation effort (e.g., design improvements, new technologies) that enhances fire safety. The expected loss with innovation effort is modelled by Eq. (9), where *b≥ 1* is a dimensionless factor reflecting the impact of the innovation effort on the expected fire-induced losses. The innovation effort is modelled as a “force multiplier” for the investment level in the sense that it increases the effectiveness of a given investment level in reducing fire losses.

9 $$D\left(C\right)=\frac{k}{bC+ a}$$

**Fig. 3:**
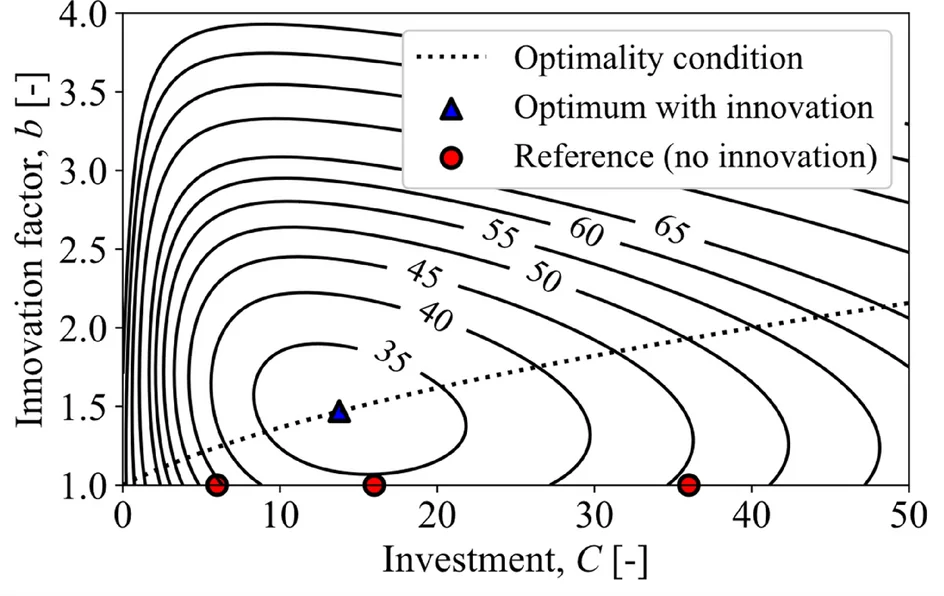
Iso-contours for the total cost of fire, in function of the investment cost and the innovation level, for $\frac{k}{a}=100$, *a=4* and ${K}_{I}=10$

The cost of innovation is modeled as: $I={{K}_{I}\left(b-1\right)}^{2}$, with ${K}_{I}$ an innovation cost constant [EUR] (e.g. [89]). Importantly, the adopted model for the innovation cost is increasing and convex, reflecting rising marginal effort or expense (i.e., innovation becomes relatively more expensive as the level of innovation increases). The total cost is given by Eq. (10). Iso-contours for the total cost of fire are visualized in Fig. 3, adopting scaled hypothetical parameter values as in Fig. 2: $\frac{k}{a}=100$, *a = 4* and ${K}_{I} = 10$. For *b=1*, the cost formulation and corresponding analysis collapse to those outlined in 4.1.1.

10 $$Z\left(C,b\right)=C+I+D=C+{{K}_{I}\left(b-1\right)}^{2}+\frac{k}{bC+ a}$$

The optimum design is defined by the pair $\left({C}_{I}^{*};{b}_{I}^{*}\right)$, i.e., the solution to the first order conditions of Eqs.(11 and 12). There is no closed form solution to these equations. The global optimum is obtained numerically as $\left({C}_{I}^{*}=13.8;{b}_{I}^{*}=1.5\right)$, corresponding with ${Z}_{I}^{*}=32.5$.

11 $$\frac{\partial Z }{\partial C}= 1-\frac{kb}{{\left(bC+a\right)}^{2}}=0$$

12 $$\frac{\partial Z }{\partial b}= 2{K}_{I}\left(b-1\right)-\frac{kC}{{\left(bC+a\right)}^{2}}=0$$

**Fig. 4:**
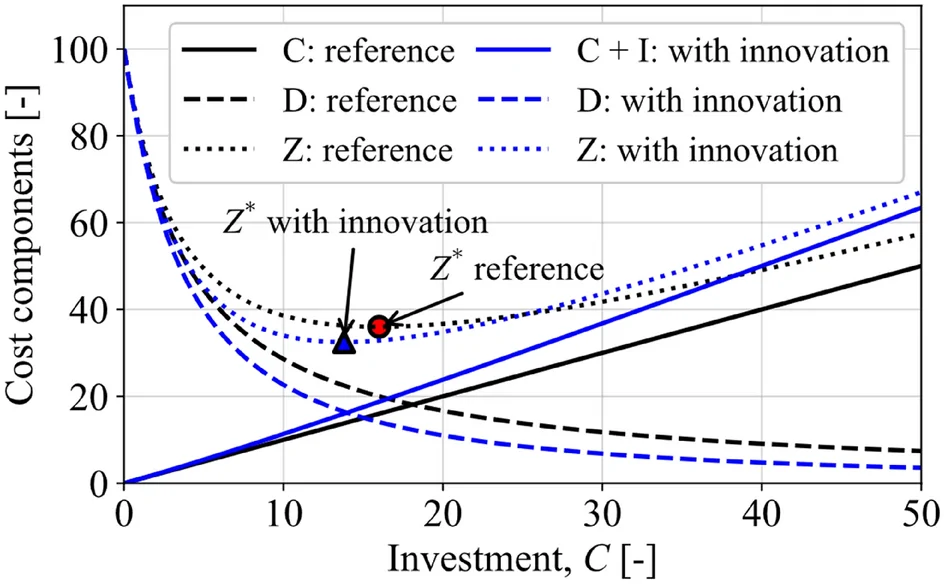
Total cost of fire, fire protection cost (investment plus innovation) and expected fire induced losses, in function of the investment cost, for $\frac{k}{a}=100$, *a=4* and ${K}_{I}=10$. Innovation level for given *b* defined by Eq. (13). Reference design in accordance with 4.1.1 (*b=1*)

The set of equations does, however, readily results in the relationship of Eq. (13), which is obtained by substituting the marginal condition for safety investment (Eq. (11)) into the first-order condition for innovation effort (Eq. (12), thereby specifying a simple relationship between the innovation effort and investment level that must hold for the optimum pair $\left({C}_{I}^{*};{b}_{I}^{*}\right)$. This relationship is also visualized in Fig. 3 (the ‘optimality condition’). Defining *b* for given *C* through Eq. (13), i.e., evaluating the cost components along the dashed line in Fig. 3, the cost components and the total cost of fire are compared in Fig. 4 with the reference results of Sect. 4.1.1 obtained without innovation (i.e., for *b=1*). Figure 4 illustrates how innovation allows the expected fire induced losses to decrease more rapidly with increasing investment *C*, but also results in a faster increase of the total fire protection cost (investment plus innovation). The evaluation with innovation results in a lower total cost of fire in the optimum point. Thus, there is an economic incentive to innovate, adopting the optimum pair $\left({C}_{I}^{*};{b}_{I}^{*}\right)$ and minimizing the total cost of fire.

13 $$C= 2{K}_{I}\left({b}^{2}-b\right)\iff b=\frac{1}{2}+\sqrt{\frac{1}{4}+\frac{C}{2{K}_{I}}}$$

The innovation effort constitutes a societally relevant cost component. If all externalities are internalized, and if no additional private cost components are considered, then the private decision-maker encounters the same optimization. Under a PBD rule which allows innovation, the adopted design aligns with the global optimum, i.e., ${C}_{PBD}={C}_{I}^{*}$ and ${b}_{PBD}={b}_{I}^{*}$. In contrast, under a strict prescriptive rule the investment level is fixed at $C={C}_{pr}$ and there is no innovation effort (${b}_{pr}=1$). Table 3 provides an overview of the DWL for the same scenarios as in Table 2, additionally considering whether innovation is allowed within the PBD rule. This analysis demonstrates how the fixed nature of prescriptive codes generates increased DWL by constraining innovation. In contrast, PBD frameworks that allow for innovation enable decision-makers to coordinate investment and innovation, thereby achieving a lower total cost of fire. A PBD rule that does not allow for innovation (e.g., by prescribing evaluation methods or design approaches rather than setting broader, outcome-oriented safety objectives) results in a DWL as the societal optimum is not achieved.

**Table 3:** Overview of the privately chosen investment level, total cost of fire and deadweight loss, considering different design rules and prescriptive guidance/requirements

Rule | Scenario	Investment, total cost, and deadweight loss	
PBD rule allowing for innovation	${C}_{PBD}^{I}={C}_{I}^{*}=13.8$ ${b}_{PBD}^{I}={b}_{I}^{*}=1.5$ ${Z}_{PBD}^{I}=32.5$ $DWL={Z}_{PBD}^{I}-{Z}_{I}^{*}=0$	
PBD rule not allowing for innovation	${C}_{PBD}^{no-I}={C}^{*}=16$ ${b}_{PBD}^{no-I}=1.0$ ${Z}_{PBD}^{no-I}=36$ $DWL={Z}_{PBD}^{no-I}-{Z}_{I}^{*}=3.5$	
Prescriptive investment	${C}_{pr}=6$	${C}_{pr}=36$
Strict prescriptive rule not allowing for innovation	${C}_{pr}^{str}={C}_{pr}=6$ ${b}_{pr}^{str}=1.0$ ${Z}_{pr}^{str}={Z}_{pr}=46$ $DWL={Z}_{pr}^{str}-{Z}_{I}^{*}=13.5$	${C}_{pr}^{str}={C}_{pr}=36$ ${b}_{pr}^{str}=1.0$ ${Z}_{pr}^{str}={Z}_{pr}=46$ $DWL={Z}_{pr}^{str}-{Z}_{I}^{*}=13.5$
Minimum prescriptive rule not allowing for innovation	${C}_{pr}^{min}={C}^{*}=16$ ${b}_{pr}^{min}=1.0$ ${Z}_{pr}^{min}={Z}_{PBD}=36$ $DWL={Z}_{pr}^{min}-{Z}_{I}^{*}=3.5$	${C}_{pr}^{min}={C}_{pr}=36$ ${b}_{pr}^{min}=1.0$ ${Z}_{pr}^{min}={Z}_{pr}=46$ $DWL={Z}_{pr}^{min}-{Z}_{I}^{*}=13.5$

#### Enforcement Costs

Enforcement costs are the (expected) administrative costs associated with enforcing the fire safety regulations. Here, enforcement costs are considered to include both oversight and (public) enforcement in the strict sense (see footnote 1). These costs thus arise either from ex ante review (i.e., assessing and approving the design before construction) or from ex post forensic investigations [90], and add to the total societal cost of fire. In what follows we treat the enforcement costs as dependent on the legal framework but independent of the chosen investment level *C.* Expanding the model of Eq. (4) from Sect. 4.1.1., the total cost of fire is given by Eq. (14), with *E* the enforcement cost.

14 $$Z\left(C,E\right)=C+D\left(C\right)+E=C+\frac{k}{C+a}+E$$

The enforcement cost under prescriptive rules is denoted ${E}_{pr}$ and typically involves straightforward upfront code compliance audits. The enforcement cost under PBD rules is denoted ${E}_{PBD}$. Many approaches to PBD enforcement are possible, ranging from self-certification to upfront third-party reviews. The enforcement cost is here considered dependent on the legal framework, but independent of *C*. The optimum investment is ${C}^{*}=\sqrt{k}-a$, as derived in 4.1.1 with Eq. (5). Adopting the same assumptions as above (externalities are internalized, no additional private cost components), the decision-maker chooses investment ${C}^{*}$ under a PBD rule. This conclusion remains valid also if the enforcement costs are not internalized. Thus, the total societal cost of fire under the PBD rule is given by Eq. (15).

15 $${Z}_{PBD}={C}^{*}+\frac{k}{{C}^{*}+a}+{E}_{PBD}$$

Under a strict prescriptive rule, investment is fixed at $C={C}_{pr}$. The total societal cost is given by Eq. (16). Under a minimum prescriptive rule, the total societal cost is given by Eq. (16) for ${C}_{pr}\ge {C}^{*}$ and by Eq. (17) for ${C}_{pr}<{C}^{*}$.

16 $${Z}_{pr}^{str}={C}_{pr}+\frac{k}{{C}_{pr}+a}+{E}_{pr}$$

17 $${Z}_{pr}^{min}={C}^{*}+\frac{k}{{C}^{*}+a}+{E}_{pr} \;for \;{C}_{pr}<{C}^{*}$$

**Fig. 5:**
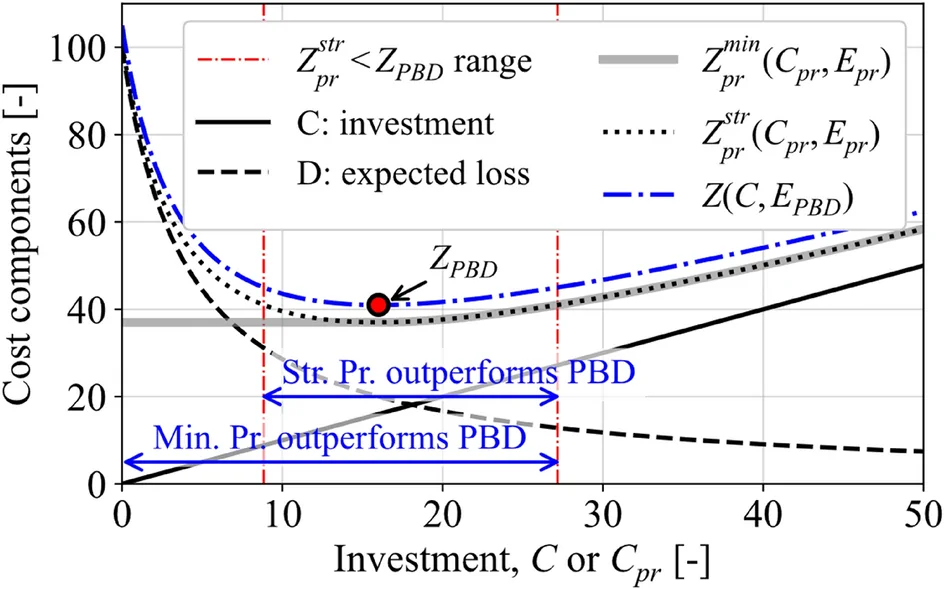
Comparison between the PBD rule and the prescriptive rules with enforcement costs. $\frac{k}{a}=100$, *a = 4*, ${E}_{PBD}=5$ and ${E}_{pr}=1$

Figure 5 illustrates how the total societal cost of fire compares between the PBD rule and the prescriptive rules. For illustration purposes, the same scaled hypothetical parameter values are considered as in 4.1.1, together with ${E}_{PBD}=5$ and ${E}_{pr}=1$. These values are chosen to make the effects visually observable. Detailed data gathering and analysis is needed in follow up research to assess current values for specific jurisdictions. The figure shows that ${Z}_{pr}^{str}$ is below ${Z}_{PBD}$ when ${C}_{pr}$ is close to ${C}^{*}$. In other words, because ${E}_{PBD}$ is in this example assumed to exceed ${E}_{pr}$, the prescriptive rule results in a lower total societal cost of fire than the PBD rule when the prescriptively required investment ${C}_{pr}$ is sufficiently close to the optimum ${C}^{*}$. In Fig. 5, the range of ${C}_{pr}$ for which ${Z}_{pr}^{str}<{Z}_{PBD}$ is indicated. The boundary points for this range are determined through Eq. (18), with solution given by Eq. (19) for completeness. In Eq. (19), ${E}_{PBD}-{E}_{pr}$ is written as *Δ E* for brevity. For the case of Fig. 5, the strict prescriptive rule outperforms the PBD rule in terms of total societal cost of fire for ${8.8<C}_{pr}<27.2$. From a practical perspective, this analysis indicates that the prescriptive requirement (i.e., ${C}_{pr}$) does not have to be precise in order to outperform the PBD rule when ${E}_{PBD}-{E}_{pr}$ is large.

18 $${Z}_{PBD}-{Z}_{pr}^{str}=0\Rightarrow 2\sqrt{k}-a-{C}_{pr}-\frac{k}{{C}_{pr}+a}+{E}_{PBD}-{E}_{pr}=0$$

19 $${C}_{pr}=\frac{\Delta E}{2}-a+\sqrt{k}\pm \frac{1}{2}\sqrt{\Delta E\left(\Delta E+4\sqrt{k}\right)}$$

The comparison between the minimum prescriptive rule and the PBD rule is also shown in Fig. 5. Under the listed assumptions, the minimum prescriptive rule outperforms the PBD rule for all ${C}_{pr}$ smaller than the higher limiting value of Eq. (19). For the example parameter values adopted in Fig. 5, this is for ${C}_{pr}<27.2$.

These analyses demonstrate that the efficiency comparison between the PBD rule and prescriptive rule (i.e., the evaluation of which rule results in the lowest total societal cost of fire) cannot be seen independent from the enforcement cost. The enforcement costs associated with different regulatory regimes are currently not clear. The analysis indicates that mapping these costs is a key area for future research. Such investigations may uncover situations where ${E}_{PBD}<{E}_{pr}$. In such situations, the PBD rule will outperform all prescriptive rules in terms of total cost of fire, as follows mathematically from Eqs.(15-17).

The analyses highlight that enforcement costs can make prescriptive rules more societally efficient than PBD rules if the prescriptive requirement is sufficiently close to the optimum (here, if ${C}_{pr}\approx {C}^{*}$). As stated earlier in Sec. 4.1.1., because buildings vary significantly in their characteristics, a uniform prescriptive investment level is unlikely to be well-suited across a wide range of contexts. Differentiating prescriptive requirements between building categories then appears a natural solution, but this would also result in an increase of ${E}_{pr}$. In the extreme, every building would have its own prescriptive requirement and ${E}_{pr}$ would be very high, at which point a simple PBD rule that by itself enables tailored investment decisions ${C}^{*}(k,a)$ will be societally more efficient. Also ${E}_{PBD}$, however, is likely to increase with increase in the PBD rule scope, e.g., because reviewers lack capacity to sustain ${E}_{PBD}$ for a large number of projects, but also because each project requires the assessment of a unique set of analyses and assumptions: a fundamentally more complex and resource-intensive task than verifying compliance with a uniform prescriptive standard that can be learned once and applied repeatedly. Extensions to the investigations presented here can then be used to evaluate whether expanding enforcement capacity is justified (i.e., results in a lower societal total cost of fire as compared to alternative scenarios such as limiting the scope of the PBD rule). Considering the above, it is expected that complementary scopes for prescriptive rules and PBD rules are most efficient overall (i.e., results in the lowest societal total cost of fire). Such complementary scopes are in effect already in place in some jurisdictions. For example, the prescriptive guidance of Approved Document B in England and Wales is limited in scope to “common building situations,”^15^ whereby a presumption of adequate safety is considered to result from application of the guidance. When the prescriptive guidance is deemed not applicable or overly restrictive (i.e., in cases of “tall, large, or complex buildings”),^16^ a (partial) PBD approach is adopted. Likewise, the new Swiss Fire Safety Regulations 2026 [91] which are scheduled to enter into force in 2027, shift the Swiss fire safety legal framework from an essentially prescriptive framework toward a structured hybrid framework that explicitly recognises prescriptive and PBD (including risk based) solutions (Bern University of Applied Sciences,2025).

### Liability Impact on Decision-Makers

The investigations in 4.1 assumed that all externalities are internalized. Under such an assumption societal fire induced losses directly translate into an economic loss for the private decision-maker, resulting in an incentive for the decision-maker to invest in safety measures. The degree to which this assumption can be considered to hold, however, depends on the legal framework, particularly with regard to legal liability. In the following, three liability approaches (i.e., Strict Liability, Negligence Based on Compliance with Code, and Negligence Based on Due Diligence) are investigated and compared in terms of (i) the total cost function for the private decision maker, (ii) the chosen investment level, and (iii) the DWL. To demonstrate key effects, a single decision-maker is considered (i.e., the fire engineer, or the building owner) and assumed to carry the investment cost *C* and the expected liability. The expected liability corresponds with the part of the fire induced losses that are internalized. The described setup thus does not consider scenarios with chains of liabilities, e.g., where building owners may claim damages from building contractors and building contractors may claim damages from engineers. To isolate the incentive structures of the liability regimes, this section focuses exclusively on the primary social costs: safety investment and expected fire loss. However, the total social cost also encompasses the administrative and enforcement costs described in Sect. 4.1.3. While these costs are omitted here to maintain analytical clarity, they may be relevant in practice; a theoretically optimal liability rule may be rendered inefficient if the costs of monitoring compliance or litigating claims outweigh its marginal safety benefits [27, 52].

#### Strict Liability

Under strict liability (hereafter, “SL”), the decision-maker is held responsible for all losses resulting from a fire, regardless of whether they followed regulations or acted reasonably. The only way for the decision-maker to avoid liability is to reduce the likelihood and/or severity of fire-induced losses. Thus, the expected liability *L* equals the expected fire-induced loss $D\left(C\right)$*.* Adopting the cost model of 4.1.1, the total cost for the private decision-maker is thus given by Eq. (20).

20 $${Z}_{SL}\left(C\right)=C+L=C+D\left(C\right)=C+ \frac{k}{C+a}$$

The model of Eq. (20) corresponds directly with the model presented earlier in Eq. (4). In technical terms, all externalities are fully internalized, and the solutions presented in 4.1.1 apply with the minimum ${Z}_{SL}$ obtained for ${C}_{SL}^{*}= \sqrt{k}-a$. The privately preferred design in case of SL thus perfectly aligns with the societal optimum described in 4.1.1.

Two important caveats are that (i) discounting and (ii) risk aversion have been ignored. Discounting results in a lower valuation of future costs compared to current costs. This reduces the optimum investment level because future fire-induced losses are valued less. If the societal discount rate and the discount rate adopted by the private decision maker are the same, then the conclusion that SL results in alignment of the societal and private optima remains valid. Private decision-makers are however likely to adopt a significantly higher discount rate than society. Effects on optimum investment levels have been shown in the literature [86, 87]. Risk aversion in turn relates to a dislike for uncertain future costs. Thus, the decision-maker may prefer to “play it safe” with proven measures. As shown by [92], such risk aversion is especially important in the context of SL, because when individuals are risk-averse, SL will lead to greater caution compared to negligence, as the perceived risk of being held liable is higher.^17^ To explore the effect of risk aversion, a risk aversion parameter $\eta \ge 0$ is introduced, modifying Eq. (20) to Eq. (21). From a societal perspective, evaluations should be done “risk neutral” [87], and thus in a societal evaluation *η =0*.

21 $${Z}_{SL}\left(C\right)=C+\left(1+\eta \right)L=C+\left(1+\eta \right)D\left(C\right)=C+\left(1+\eta \right) \frac{k}{C+a}$$

The first order condition is given in Eq. (22), resulting in the optimum investment level for the risk averse private decision-maker ${C}_{SL}^{*}=\sqrt{ \left(1 +\eta \right)k} - a$. If *η =0*, ${C}_{SL}^{*}$ reduces to ${C}^{*}$ and the privately chosen investment level aligns with the societal optimum. In this case, the SL regime does not result in a DWL. If *η >0*, the private decision-maker experiences an incentive to overinvest beyond the societal optimum, i.e., ${C}_{SL}^{*}>{C}^{*}$. For completeness, the DWL is given by Eq. (23), where ${Z}_{SL}\left(C\right)$ represents the total cost function of the private decision-maker under SL, while $Z\left({C}_{SL}^{*}\right)$ and ${Z}^{*}$ represent the societal total cost of fire evaluated at the privately chosen investment level and at the societal optimum, respectively.

22 $$ \frac{{dZ_{SL} }}{dC} = 1 - (1 + \eta )\frac{k}{{(C + a)^{2} }} = 0 \Rightarrow C_{SL}^{ * } \, = \sqrt {\left( {1 + \eta } \right)} k - a $$

23 $${DWL}_{SL}={Z\left({C}_{SL}^{*}\right)-Z}^{*}=\sqrt{k}\left(\frac{2+\eta }{\sqrt{1+\eta }}-2\right)$$

#### Code Compliance Negligence

**Fig. 6:**
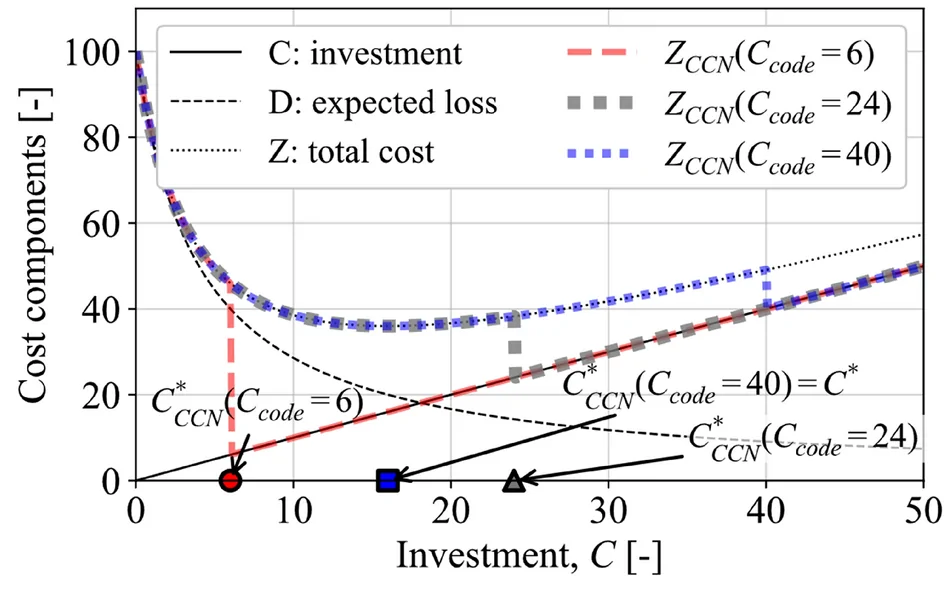
Investment cost, fire-induced losses, total societal cost of fire $Z\left(C\right)$ and private total cost of fire${Z}_{CCN}\left({C,C}_{code}\right)$, in function of the investment cost, for$\frac{k}{a}=100$,*a = 4*, and selected ${C}_{code}$ values: 6, 24,40. Scatter plot indicates the investment level chosen by the private decision-maker${C}_{CCN}^{*}$, together with corresponding *Z* and ${Z}_{CCN}\left({C,C}_{code}\right)$

Under Code Compliance Negligence (hereafter, “CCN”), decision-makers are not liable if their safety investment exceeds code requirements (reflecting the philosophy that decision-makers should not be held responsible for precautions that the law does not explicitly mandate; [51]). Within the model adopted here, this means no liability when the investment level exceeds the required minimum level, ${C}_{code}$. If the investment falls below the threshold, the decision-maker is deemed negligent and liable for the full loss if a fire occurs. The expected cost function is given by Eq. (24) and visualized in Fig. 6 for selected scenarios. Note that ${C}_{code}$ does not necessarily equal ${C}_{pr}$, and that the design rules (prescriptive or PBD) can in principle be combined with any of the liability regimes.

24 $${Z}_{CCN}\left(C\right)=\left\{\begin{array}{c}C\\ C+L=C+ D\left(C\right)\end{array}\right. \genfrac{}{}{0pt}{}{{for\; C\ge C}_{code, }}{{for \;C<C}_{code.}}$$

If the code-mandated investment level ${C}_{code}$ is below the unconstrained (societal) optimum investment level ${C}^{*}$, then under CCN, the optimum for the private decision-maker is to invest only up to the mandated level, i.e., ${{C}_{CCN}^{*}=C}_{code}$. Any larger *C* raises cost without further liability reduction. This scenario is illustrated for ${C}_{code}=6$ in Fig. 6 and results in an underinvestment relative to the societal optimum. The decision-maker meets ${C}_{code}$ to avoid liability, but has no incentive to exceed it, even if additional investment could further reduce the total societal cost of fire. The conclusion ${{C}_{CCN}^{*}=C}_{code}$ also holds for ${C}_{code}>{C}^{*}$, as long as ${C}_{code}\le Z\left({C}^{*}\right)$. Considering Eq. (24), the total cost of fire for the private decision-maker is smallest when investing ${C}_{code}$, even though it exceeds the unconstrained optimum ${C}^{*}$. This scenario is illustrated in Fig. 6 for ${C}_{code}=24$ and results in an overinvestment relative to the societal optimum. The private decision-maker minimizes costs by escaping liability. Only when ${C}_{code}$ becomes very large does the cost of the mandated overinvestment outweigh the private benefit of avoiding liability. In that case, ${C}_{CCN}^{*}={C}^{*}$, as illustrated in Fig. 6 for ${C}_{code}=40$. In mathematical terms, and taking into account the solution for ${Z}^{*}$ given in Eq. (6), the investment level chosen by the private decision-maker is given by Eq. (25).

25 $${C}_{CCN}^{*}=\left\{\begin{array}{c}{C}_{code}\\ {C}^{*}\end{array}\right. \genfrac{}{}{0pt}{}{for \;{C}_{code}\le {Z}^{*}=2\sqrt{k}-a}{for \;{C}_{code}>{Z}^{*}=2\sqrt{k}-a}$$

**Fig. 7:**
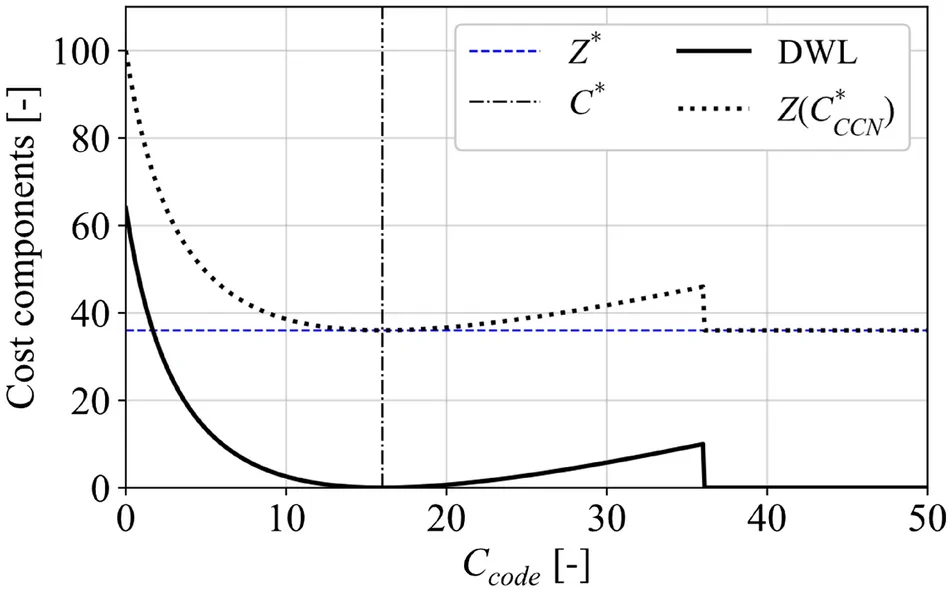
Total societal cost of fire under the CCN rule, $Z\left({C}_{CNN}^{*}\right)$, societal optimum ${Z}^{*}$ and DWL, in function of ${C}_{code}$

Taking into account Eqs.(4) and (6), the DWL associated with CCN, i.e., $Z\left({C}_{CCN}^{*}\right)-{Z}^{*}$, is given by Eq. (26). This is illustrated by Fig. 7. Although there is a non-zero DWL for all ${C}_{code}$ in the vicinity of ${C}^{*}$, the DWL changes slowly near the optimum design. For the model assumptions adopted here, this change is furthermore slower for mandated overinvestment (i.e., ${C}_{code}>{C}^{*}$). Additionally, when ${C}_{code}$ far exceeds the optimum, the DWL returns to zero as the private decision-maker is incentivized to deviate from the excessive code mandated investment. Together, these observations suggest that (i) a CCN regime can be efficient when ${C}_{code}$ approximates the optimum investment level for a category of buildings; (ii) it is recommendable to err on the side of larger ${C}_{code}$ in case of doubt or when rounding optima for implementation as code requirements; and (iii) a CCN regime whereby ${C}_{code}$ entails a major overinvestment for specific categories of buildings does not necessarily constitute a problem. To clarify the last point with an example, consider a blanket rule R90 fire resistance requirement for warehouses. This requirement is likely to constitute a major overinvestment for warehouses with very limited fire load (e.g., storage of non-combustible goods). Under the CCN regime, DWL could nevertheless be avoided since the private decision-maker is incentivized to optimize the structural fire protection and forego the liability exclusion associated with an R90 design.

26 $${DWL}_{CCN}=\left\{\begin{array}{c}{C}_{code}+\frac{k}{{C}_{code}+a}-2\sqrt{k}+a\\ 0\end{array}\right. \genfrac{}{}{0pt}{}{for \;{C}_{code}\le 2\sqrt{k}-a}{for \;{C}_{code}>2\sqrt{k}-a}$$

#### Due Diligence Negligence

**Fig. 8:**
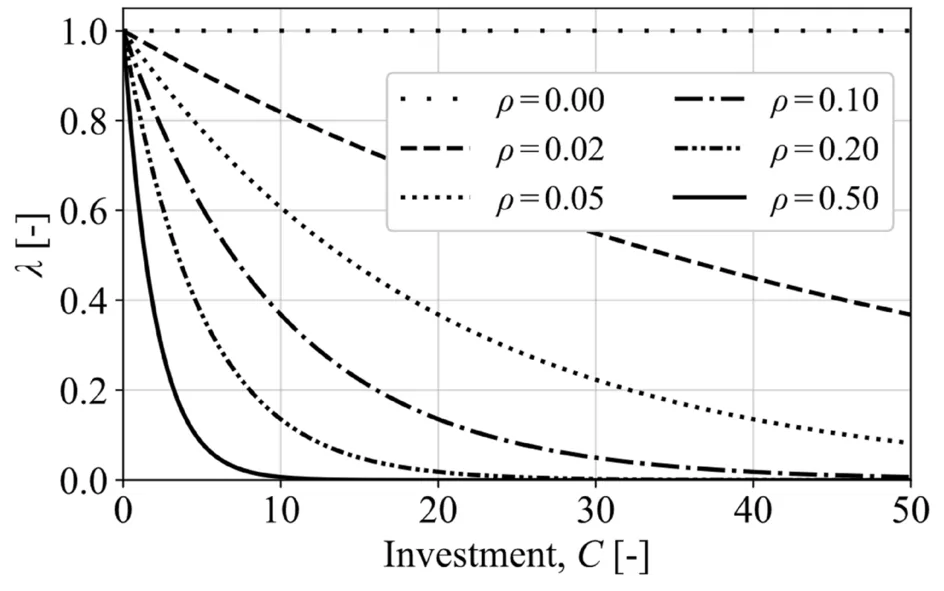
Probability *λ * of being found negligent in function of the investment level *C*, for different ρ values

Under Due Diligence Negligence (hereafter, “DDN”), liability depends on whether the decision-maker exercised reasonable care, as judged by professional standards rather than a fixed threshold. Here, courts assess liability based on whether the decision-maker exercised the care of a reasonably skilled professional [66]. In such conditions, the decision-maker trades off safety investment against a gradual reduction in both expected losses resulting from fire incidents and liability risk. The total cost of fire for the private decision maker is given by Eq. (27). The fire-induced losses are only internalized to the degree that the decision-maker is found liable. This effect is captured in Eq. (27) through the probability *λ (C)* of being found negligent. This probability reduces with investment level *C*. For illustration purposes, $\lambda (C) = {e}^{-\rho C}$ is adopted, with $\rho \ge 0$. If *ρ =0*, then there is liability irrespective of the investment level (i.e., $\lambda \left(C,\rho =0\right)=1$), and the model is then equivalent to a case of strict liability (without risk aversion). For *ρ > 0*, a higher effort *C* results in a reduced probability of being found negligent. The higher *ρ *, the faster *λ * decreases with increasing investment level. This is illustrated in Fig. 8.

27 $${Z}_{DDN}\left(C\right)=C+L=C+D\left(C\right)\lambda \left(C\right)=C+\frac{k}{C + a}{e}^{-\rho C}$$

**Fig. 9:**
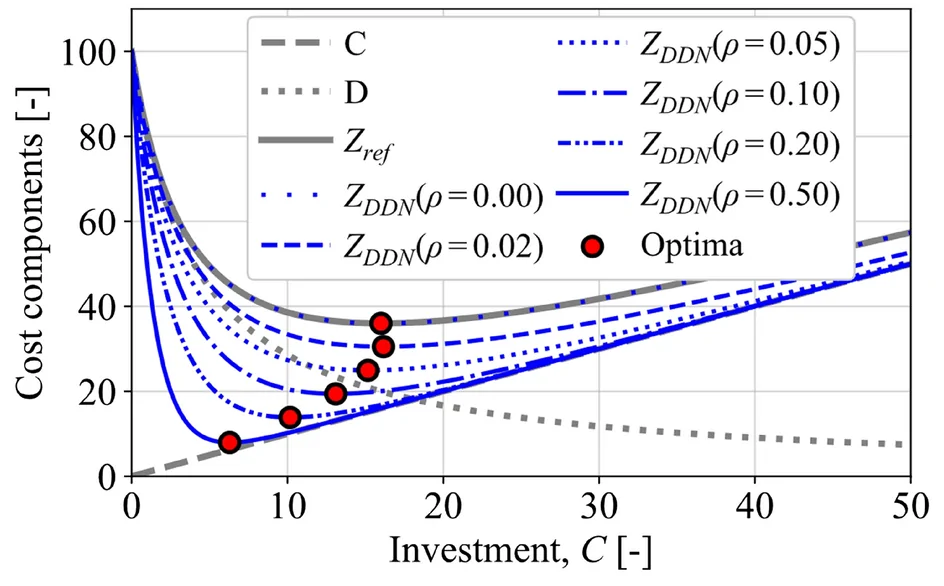
Investment cost, fire-induced losses, total societal cost of fire $Z\left(C\right)$ and private total cost of fire ${Z}_{DDN}\left(C,\rho \right)$, in function of the investment cost, for $\frac{k}{a}=100$, *a = 4*, and selected *ρ * values. Scatter plot indicates the investment level chosen by the private decision-maker ${C}_{DDN}^{*}$, together with corresponding ${Z}_{DDN}\left(C,\rho \right)$

The first order condition is given by Eq. (28). There is no easy closed form solution, and the optimum investment level ${C}_{DDN}^{*}$ for the decision maker is determined numerically. Results are visualized in Fig. 9 together with the total cost function of Eq. (27). In this figure, ${Z}_{ref}$ indicates the case of strict liability (without risk aversion; see Sec. 4.2.1). For *ρ =0*, ${C}_{DDN}^{*}={C}^{*}$, and there is no DWL. For small *ρ *, ${C}_{DDN}^{*}\approx {C}^{*}$ and the DWL is very small. With increasing *ρ *, ${C}_{DDN}^{*}$ decreases because the decision-maker has a low probability of being held liable (i.e., the fire induced losses are only internalized to a limited extent), and the DWL increases. The DWL for the scenarios listed in Fig. 9 is given in Table 4. The results show that DDN very effectively limits the DWL when the burden for proving reasonable care is sufficiently high (i.e., when *ρ * in the model is not very large). For the given model assumptions, all significant deviations between ${C}_{DDN}^{*}$ and ${C}^{*}$ constitute an underinvestment.

**Table 4:** Overview of the privately chosen investment level, total cost of fire and deadweight loss, considering different *ρ * value

Scenario	${C}_{DDN}^{*}$	$D\left({C}_{DDN}^{*}\right)$	$\lambda \left({C}_{DDN}^{*}\right)$	${Z}_{DDN}\left({C}_{DDN}^{*},\rho \right)$	$DWL=Z\left({C}_{DDN}^{*}\right)-{Z}^{*}$
*ρ =0.00*	16.00	20.00	1.00	36.00	0.00
*ρ =0.02*	16.16	19.84	0.72	30.52	0.00
*ρ =0.05*	15.16	20.88	0.47	24.94	0.04
*ρ =0.10*	13.10	23.39	0.27	19.41	0.49
*ρ =0.20*	10.17	28.23	0.13	13.86	2.40
*ρ =0.50*	6.29	38.87	0.04	7.96	9.16

28 $$\frac{{dZ}_{DDN}\left(C\right)}{dC}= 1- \frac{k}{C + a} {e}^{-\rho C}\left(\frac{1}{C+a}+\rho \right)=0$$

#### Comparison of Liability Regimes

As demonstrated through the investigations above, the liability regime critically shapes the behavior of decision-makers in fire safety. In particular, it influences how much they invest in risk mitigation. The main conclusions of the liability regime investigations are summarized as:

- **Strict Liability** fully internalizes the fire-induced losses, thereby aligning private and societal investment incentives. If the private decision-maker is risk averse, however, the private decision-maker experiences an incentive to overinvest beyond the societal optimum.
- **Code Compliance Negligence** simplifies enforcement and ensures minimum safety standards by requiring compliance with a legally fixed threshold, ${C}_{code}$. This approach however introduces a discontinuity in the total expected cost function for the private decision maker. Liability for the fire-induced losses is incurred in case of investments below ${C}_{code}$, while investments beyond ${C}_{code}$ yield no liability benefit. This “cliff effect” distorts incentives, leading to over- or under-investment. The DWL is only avoided when ${C}_{code}$ exceeds the optimum total fire cost ${Z}^{*}={C}^{*}+D\left({C}^{*}\right)$, or in the theoretical case where ${C}_{code}$ perfectly matches the societal optimum investment ${C}^{*}$. Moreover, if the prescriptive requirements are well-calibrated such that ${C}_{code}$ is close to ${C}^{*},$ the resulting DWL remains reasonably low, and the regime may be relatively 'efficient' given its lower administrative and verification costs. This is reflected in the numerical results in Table 5, where the efficiency of this regime improves as the compliance threshold approaches the optimum.
- **Due Diligence Negligence** introduces a smooth liability gradient whereby higher *C* progressively lowers both fire-induced losses and the probability of being deemed negligent (i.e., the probability of having to pay for the fire losses). This approach effectively pushes the private decision-maker towards the optimum investment level, as long as the negligence probability does not decrease fast with increasing *C*.

**Table 5:** Comparison of liability regimes

Aspect	Strict Liability (SL)	Code Compliance Negligence (CCN)	Due Diligence Negligence (DDN)
Liability (for decision-maker)	Always liable for actual losses	Liable only if $C<{C}_{code}$	Liable if investment falls below standard of care
Risk Allocation	Full burden on decision-maker	Risk shifted to others once code requirement is met. Creates moral hazard if ${C}_{code}$ is set too low (i.e., decision-makers have no incentive to exceed the minimum standard and will underinvest if ${C}_{code}<{C}^{*}$	Risk shared dynamically based on standard of care (professional standards based on state of the art)
Private optimum investment level	Aligns with societal optimum ${C}^{*}$	Private decision-makers invest to ${C}_{code}$, also if ${C}_{code}$ exceeds the societal optimum ${C}^{*}$. The adopted investment shifts to ${C}^{*}$ only when ${C}_{code}$ exceeds the optimum total cost of fire ${Z}^{*}$	Tends towards societal optimum ${C}^{*}$. Underinvestment in case of low reasonable care threshold
Efficiency (inverse of deadweight loss)	High under ideal conditions (*DWL=0*). Risk aversion, however, results in overinvestment	Variable. Efficient when ${C}_{code}\approx {C}^{*}$. DWL increases with increasing difference between ${C}_{code}$ and ${C}^{*}$, up to the point where ${C}_{code}$ exceeds the optimum total cost of fire, in which case DWL returns to zero	High, with *DWL≈ 0* when there is a strong standard of care
Innovation Incentive (hypothesis)	Low due to assumed higher risk aversion with regard to new design approaches	Very low: deviation from code risks non-compliance and liability	High: innovation allowed if demonstrably safe and professionally justifiable

A structured comparison between the liability regimes is provided in Table 5, including a preliminary discussion on innovation incentives. Future work should extend the model applications to consider the interaction between design rules (prescriptive or PBD) and liability regimes, take into account insurance effects and chains of liability, and explicitly model time effects (discounting).

## Conclusion

The application of L&E methodologies to fire safety offers a structured approach to remedy regulatory failures and to ensure that a societally optimal level of fire safety is achieved. In this paper, fundamental concepts of L&E have been introduced, together with a basic model for the economic analysis of legal frameworks around fire safety. The model has been applied to reveal how liability rules and regulatory design shape incentives and influence stakeholder behavior. When considering full internalization of all fire-related costs (i.e., when the decision maker carries the full burden of fire), the model demonstrates that rigid prescriptive requirements necessarily lead to over- or under-investment in preventive measures, resulting in a total societal cost of fire exceeding the optimum. PBD approaches, on the other hand, incentivize decision makers to adopt societally optimal safety investments. The degree to which fire-related costs are internalized, however, depends on the liability regime. Where fire-related costs are not internalized, a strong incentive to underinvest in safety may exist. This is notably the case for a liability regime of code compliance negligence (i.e., a liability regime whereby decision-makers avoid all liability when meeting code requirements), if the code-prescribed safety investment is below the societal optimum level.

The obtained insights can be leveraged by policymakers and standard‐setting bodies to balance safety objectives, innovation, and cost constraints. Individual fire engineers can also draw on the underlying economic logic, e.g., when documenting their professional judgments (“due diligence”) to manage liability risk, and by using simple cost–benefit reasoning when evaluating PBD solutions.

While this article lays the conceptual groundwork, several avenues merit further exploration: (1) The development of case studies illustrating the application of L&E integration in real-world settings, offering practical illustrations to guide both policymakers and practitioners; (2) Empirical validation of the model’s predictions, including a calibration of model parameters such as enforcement costs on real-world data; (3) Analysis of how insurers price fire risk under differing regulatory regimes to reveal additional market-based levers for safety promotion; (4) Expansion of the model to consider multiple actors and their interaction (e.g., chains of liability); (5) Comparative analysis of different legal systems (e.g. common law versus civil law traditions) to clarify their influence on legal frameworks for fire safety and the potential benefit of harmonization across jurisdictions; (6) Specifying, refining and validating a proposal for a new legal framework, as inspiration for policymakers.

## Data Availability

No datasets were generated or analysed during the current study.

## Footnotes

1 In this context, “enforcement” is understood broadly to include both oversight and sanctioning. Oversight includes the proactive monitoring and verification of compliance (e.g., through design reviews, third-party audits, and site inspections), whereas sanctioning involves the application of sanctions to compel adherence or address breaches [16]. This includes public enforcement via administrative or legal penalties (e.g., fines, stop-work orders, or prosecution) and private enforcement through civil damage claims intended to compensate victims and (possibly) punish non-compliance [16, 18].

2 Yet, a certain degree of complexity can be deliberate, effectively signaling when mainstream contractors should cede to specialized firms.

3 While this article adopts an L&E lens to formalize these relationships, it is acknowledged that L&E is one of several competing frameworks for evaluating law. Other frameworks include e.g. legal philosophy, ethics, and law and psychology (e.g. [24]). This paper does not claim that L&E is the only valid perspective,rather, it seeks to demonstrate the specific utility of an L&E approach in aligning fire safety engineering practices with legal incentives and societal welfare objectives.

4 While market failures provide the justification for legal intervention [26, 27], it is also important that the legal mechanisms themselves are well-designed so that they actually generate the intended societal benefits. For example, Australian studies have shown that harmonizing fragmented building codes, moving toward PBD approaches, and reducing unnecessary regulatory differences have lowered compliance costs, stimulated innovation, and improved overall economic efficiency without compromising safety objectives [28, 29]

5 Cook and Taylor [14] illustrate this dynamic in the context of combustible cladding scandals, where developers took advantage of buyers’ limited knowledge to install unsafe materials. Law and Spinardi [56] add that the Grenfell Inquiry exposed how reliance on expert intermediaries deepened informational asymmetries, leaving regulators ill-equipped to evaluate conflicting safety claims and highlighting the need for clearer legal mandates.

6 In this regard, Van den Bergh & Visscher [18] describe safety itself as “a school book example of a public good, even though it may be privatized by offering protection only to people paying for the safety services (club goods).” Hence, “also legal rules on safety have characteristics of public goods” [18].

7 There are even studies indicating safety regulations with a net disbenefit to society (e.g.. [35, 36]).

8 Some legal scholars argue that certain areas, including healthcare and potentially fire safety, should not be assessed solely on utilitarian or economic grounds [40]. From this perspective, the monetization of life and safety risks may risk “corrupting” important social values by reducing human life to an economic variable rather than treating it as a value that warrants equal respect regardless of cost [40]. It must however be highlighted that the reduction of risk to life is monetized, not life itself [41]. Nevertheless, while this article focuses on L&E, with therefore economic efficiency as an analytical lens, a comprehensive legal framework should also consider broader non-economic and rights-based considerations (see e.g., [35]).

9 In this regard, the L&E literature highlights the distinction between Common Law and Civil Law systems, often referred to as the “legal origins theory.” We note that, generally, Civil Law systems are more prescriptive [42]. For further discussion of the legal origins theory topic, we refer to Korsten & Samuel [42].

10 E.g., Belgian law requires all construction professionals (including contractors, architects, and engineers) to obtain insurance coverage for their professional liability under Articles 1792 and 2270 of the Civil Code and the Law of 9 May 2019 on Compulsory Professional Liability Insurance in the Construction Sector, provided that the services relate to the structural components of the building.

11 In practice, however, most designs are done considering prescriptive guidance, such as those listed in Approved Document B, whereby a presumption of adequate safety is considered to result from application of the guidance. Only when the prescriptive guidance is deemed not applicable or overly restrictive, a (partial) PBD approach is adopted.

12 Moral hazard refers to the individual tendency to take on greater risks when actors do not bear the full costs of their actions. In the context of (fire) insurance, this means that insured parties may reduce their investment in preventive measures because they expect the insurer to cover losses in the event of a fire, which can undermine overall safety [69].

13 We note that in practice, decision-makers may deviate from purely rational behavior due to among others cognitive biases, bounded rationality, and other behavioral factors [38, 39], as discussed in Sect. 2.1.4 and further recognized in Sect. 3. The insights derived from rational choice theory should therefore be understood as indicative of the direction of incentive effects rather than precise predictions of individual behavior [30, 39].

14 A notable exception is an RIA study by the government of New Zealand’s on shifting from joint-and-several to proportionate liability in the building and construction sector [84].

15 Section 0.9, Approved Document B: Fire Safety, Volume 1 – Dwellinghouses and Sect. 0.9, Approved Document B: Fire Safety, Volume 2 – Buildings Other Than Dwellinghouses.

16 Introduction (page iii), Approved Document B: Fire Safety, Volume 1 – Dwellinghouses and Introduction (page iii), Approved Document B: Fire Safety, Volume 2 – Buildings Other Than Dwellinghouses.

17 For the same reason, we do not examine risk aversion under the negligence rules in Sects. 4.2.2 and 4.2.3., as we consider that doing so would be redundant for the purposes of this article.
